# Anodic Desulfurization of Heterocyclic Thiones – A Synthesis to Imidazoles and Analogues

**DOI:** 10.1021/acs.orglett.4c03413

**Published:** 2024-10-28

**Authors:** Davide Cesca, Philip Arnold, Dainis Kaldre, Fabio Falivene, Filippo Sladojevich, Kurt Puentener, Siegfried R. Waldvogel

**Affiliations:** † Department of Chemistry, Johannes Gutenberg University, Duesbergweg 10−14, 55128 Mainz, Germany; ‡ Department of Process Chemistry & Catalysis, F. Hoffmann-La Roche Ltd, 4070 Basel, Switzerland; § Pharma Research and Early Development, Roche Innovation Center Basel, F. Hoffmann-La Roche Ltd, 4070 Basel, Switzerland; ∥ Department of Electrosynthesis, Max-Planck-Institute for Chemical Energy Conversion, Stiftstrasse 34-36, 45470 Mülheim an der Ruhr, Germany; ⊥ Karlsruher Institut für Technologie, Kaiserstraße 12, 76131 Karlsruhe, Germany

## Abstract

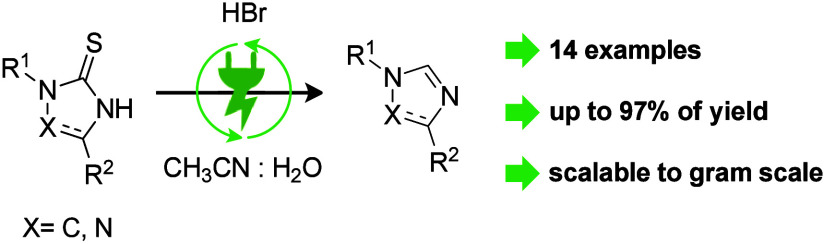

An electrochemical desulfurization of 2-mercapto-imidazoles to the corresponding imidazole is established. This novel anodic transformation is bromide-mediated and easy to conduct in the simplest electrochemical setup, consisting of an undivided cell, carbon electrodes, and constant current electrolysis. The method proved successful in 14 diverse examples of imidazoles and triazoles with up to a 97% yield. The scalability was proven in the multigram synthesis of a technically relevant N-heterocyclic carbene (NHC) ligand precursor.

Nitrogen-containing molecules, especially imidazoles, are highly significant in heterocyclic chemistry due to their diverse chemical properties and applications in agriculture and pharmacology.^1−3^ Imidazole cores are crucial in biologically active compounds because they interact well with various enzymes and receptors.^4^ Imidazolium salts are versatile and widely used as precursors for NHC ligands.^5^ Imidazole is also the third most common structure on the WHO list of essential medicines.^6^ Several methods exist for forming imidazole scaffolds.^7,8^ However, these methods often suffer from low yields, hazardous chemicals, and harsh conditions. The Ullman reaction is another method for N-functionalizing imidazoles, but it requires a homogeneous metal catalyst and elevated temperatures.^9^ A more convenient method for synthesizing imidazole is the desulfurization of 2-thione derivatives, which are either commercially available or easily synthesized by the Marckwald reaction,^10^ offering an alternative to traditional approaches, potentially overcoming some of their significant drawbacks.

The desulfurization reaction can be performed via the reduction or oxidation of the C–S bond (Scheme 1). Reductive desulfurization relies on Raney nickel, which poses safety risks due to its pyrophoric nature and challenges in disposal.^11,12^ Oxidative desulfurization requires harsh and hazardous reagents like peroxides.^13^ Baxendale et al. solved this issue by oxidizing imidazole-2-thione in flow by an in-situ generated nitrosonium agent.^14^

**Scheme 1:**
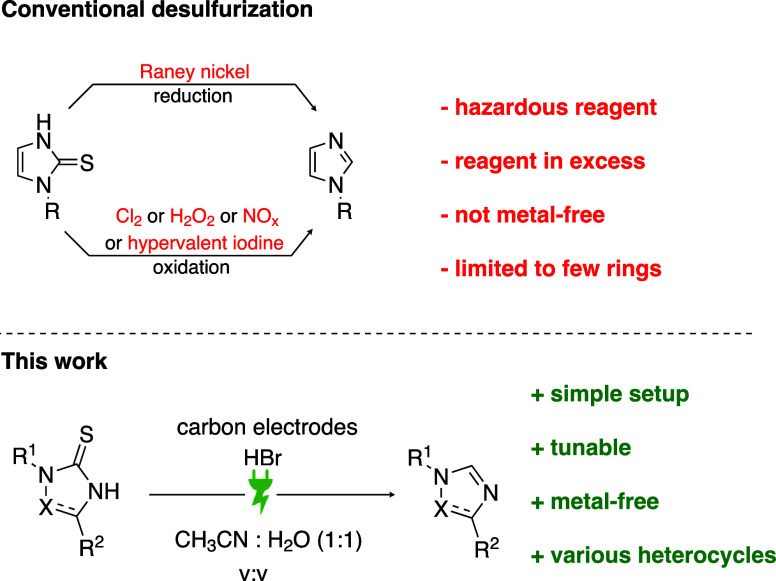
Approaches to the Desulfurization of Imidazole-2-thiones

Another major issue of oxidative desulfurization is the lack of a standard protocol; different oxidants can lead to different products. This dependence on the combination oxidant/substrate makes this transformation less appealing and rarely employed.^13^

In the 21st century, a renewed interest in electrosynthesis due to the need for greener synthetic processes and innovative reactivities occurred.^15−19^ Electrosynthesis is valued for its safety, such as generating hazardous chemicals on demand (in-cell).^20^ This makes organic electrosynthesis a central tool for future developments. However, cost-efficient downstream processing is essential for practical applications.^21^

The anodic desulfurization will generate the oxidizer required in-cell, minimizing the issues associated with handling and storage. Despite electrochemical oxidation being intensively studied and applied for the synthesis of the S=O moiety,^22−24^ reports about desulfurization are scarce. Zhu et al. reported the anodic oxidation of thioureas to the sulfate, making the carbon prone to nucleophilic attack by alkoxides and amines.^25^ Following reports showed the use of hypervalent iodine complex for synthesis of guanidines.^26,27^

This study introduces a new method for the anodic desulfurization of 2-mercaptoimidazole using in-cell electrochemical generation of bromine species. This approach eliminates risks associated with storing large amounts of corrosive bromine and improves atom economy and cost-efficiency. The reaction conditions were optimized using 4-chlorophenyl-N-imidazole-2-thione (**2b**), synthesized via the Marckwald method (see SI). The screening^28^ (Table 1), used a 1:1 mixture of acetonitrile and water, with water serving as a proton source for the cathodic hydrogen evolution reaction (HER), in order to avoid the reduction of the product.^29^ The experiments were conducted in undivided cells, emphasizing the choice of electrode for the counter-reaction to enable practical electrosynthesis.^30,31^

**Table 1:**
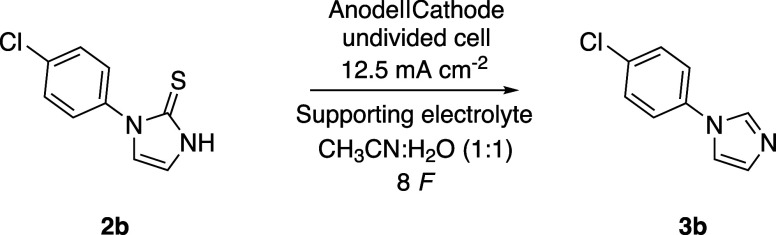
Screening of the Conditions for the Oxidative Desulfurization of 1-(4-Chlorophenyl) Imidazole-2-thione

Entry	Electrodes	Supporting electrolyte	Yield (%)^a^
1	C_(g)_||C_(g)_	HBF_4_ (10 equiv)	9
2	C_(g)_||C_(g)_	NaOH (10 equiv)	n.d.
3	C_(g)_||C_(g)_	HCl (10 equiv)	53
4	C_(g)_||C_(g)_	HBr (10 equiv)	87
5	C_(g)_||C_(g)_	HI (10 equiv)	5
6	C_(g)_||C_(g)_	NaBr (10 equiv)	80
7	C_(g)_||C_(g)_	NaBr (10 equiv) AcOH (10 equiv)	61
8	Sigraflex||C_(g)_	HBr (10 equiv)	74
**9**	**GC||C**_(g)_	**HBr (10 equiv)**	92 (90)^b^
10	GC||stainless steel	HBr (10 equiv)	93
11	GC||C_(g)_	HBr (5 equiv)	92
12	GC||C_(g)_	HBr (1 equiv)	93
13	GC||C_(g)_	HBr (0.5 equiv)	94

a Yield of **3b** was determined by ^1^H NMR spectroscopy using 1,3,5-trimethoxybenzene as internal standard.

b Isolated yield

Initially, the reaction was conducted with HBF_4_ (Table 1, entry 1) or NaOH (entry 2), which are inert supporting electrolytes. The poor yield obtained led to the rationale that the substrate cannot go under direct anodic desulfurization and a dual-role electrolyte is needed.^32^ Involving HCl (entry 3) or HBr (entry 4) increased the yield to 53% and 87%, respectively. A poor yield was obtained using HI (entry 5), due to the lower oxidative power of the species generated. The high yield achieved with HBr prompted an attempt to substitute the bromide source (entry 6). Comparing the results with NaBr and HBr (entries 6 and 4) indicated that acidic media improves yield. The plausible rationale might be the generation of radical or the diatomic species, the dominant oxidizing species at low pH.^33^ The pH of the run with NaBr (entry 6) started to rapidly increase around 4 F, reaching pH 1 toward the end of the reaction. Using an equimolar mixture of NaBr and acetic acid lowered the yield (61%), most likely due to competition between the oxidation of the bromine and the oxidation of the acetate. The higher yield achieved with HBr (87%) compared to NaBr (80%) suggests that radicals are the most active species for oxidation. This is further supported by the lower yields with HCl, as Cl radicals have a higher rate of mineralizing organic molecules.^34^ Next, the anodic material was screened (entries 7–9). Glassy carbon (GC) had a significantly higher yield compared to other carbon-based electrodes. This could be attributed to different carbon structures on the surface influencing electron transfer. On the contrary, the nature of the cathode does play a minor role, since the hydrogen evolution is sufficiently facilitated (entries 9 and 10). Also, the reaction is not strongly dependent on the amount of mediator used, as different amounts gave comparable yields of 87–94% (entries 9, 11–13).

According to the literature, oxidation using bromide as a mediator should be catalytic.^35,36^ Since the results indicated by entries 9 and 13 have comparable yields (92% and 94%, respectively), it was interesting to explore the extremes of the system and further experiments with substoichiometric and overstoichiometric amounts of mediator. The complete list of the performed experiments is given in the SI. Further screening with 0.5 equiv of mediator indicated that at a current density of 12.5 mA cm^–2^ the optimum between oxidation of the substrate and regeneration as mediator is achieved. Interestingly, when 10 equiv of HBr was employed, the system tolerated higher concentrations and current densities. This tolerance can be attributed to the larger amount of bromide present, preventing overoxidation of the product. It is noteworthy that with a current density of 12.5 mA cm^–2^, upon workup, transparent crystals were obtained, while higher current density resulted in more impurities, preventing the product from directly crystallizing. Next, we tested the reported condition on other N-substituted imidazoles. The runs with the different substrates were conducted using two conditions: 0.5 equiv of HBr and 10 equiv of HBr (see SI Table S4 for all experiments). The results revealed an interesting relationship between substrate and equivalents of mediator. We observed that for **3b** and **3c**, both conditions work well with 0.5 and 10 equiv, resulting in yields of 90% for **3b** and 84% for **3c**, respectively (Figure 1), while, for the 4-chlorophenyl substituted **3a** we obtained a better yield with 10 equiv of HBr (84% yield). Also, **3f** showed the same yield in both conditions, 71% yield. Entries **3d** and **3e** have higher yields with 10 equiv (84% and 90%, respectively) compared to 0.5 eq. With electron-releasing groups (**3g**, **3h** and **3l**), we observed a better yield with 10 equiv compared to that obtained with 0.5 equiv (92%, 87%, and 97%, respectively). With the cyclohexyl substituted **3j** we obtained a better yield with 0.5 equiv instead of 10 equiv; the rationale for this can rely on the abstraction of the hydrogen atom alpha to the substituted nitrogen by the bromine radical. This point confirms the radical species as the main oxidant. Also, triazoles (**3k**–**3m**) were explored. Testing the anodic desulfurization with triazole derivatives, we observed a higher dependency between the substrate and the HBr equivalent compared to the imidazole derivates. All the triazole tested showed a moderate yield with 0.5 equiv (26–51% yield, Table S4, SI) whereas with 10 equiv a significant increase up to 53–89% yield was found (Table S4, SI). This suggests some kind of ionic interaction between the protonated product in solution and the counteranion. This idea was tested using 1 equiv of HBr and the yield obtained was almost the same as that with 10 equiv. An observed outlier was when compound **3k** was applied; using 1 equiv of HBr, we had a better yield (79% yield) compared to 10 equiv (53% yield), most likely because a higher concentration of Br radicals or BrO_*x*_ species degraded the molecule faster. Another behavior of the system is found using benzimidazole (**3r**); a good yield (80%) was obtained with 10 equiv while with 0.5 equiv of HBr minimal conversion and degradation of the starting material was observed. The reason for this relies on the anodic degradation of the substrate at low concentration of HBr while at higher concentrations the HBr is acting as anodic protection. Some limitations were found: dimethyl-pyrimidine-2-thione (**3o**) dimerized through the sulfur–sulfur bond while *N,N*-phenyl urea 2-thione (**3p**) and thiobarbituric acid (**3q**) decomposed due to the acidic conditions. In the case of **3n**, the runs with 10 and 0.5 equiv of HBr did not give full conversion due to the poor solubility of the compound. The best yield achieved for compound **3n** was 67%.

**Figure 1:**
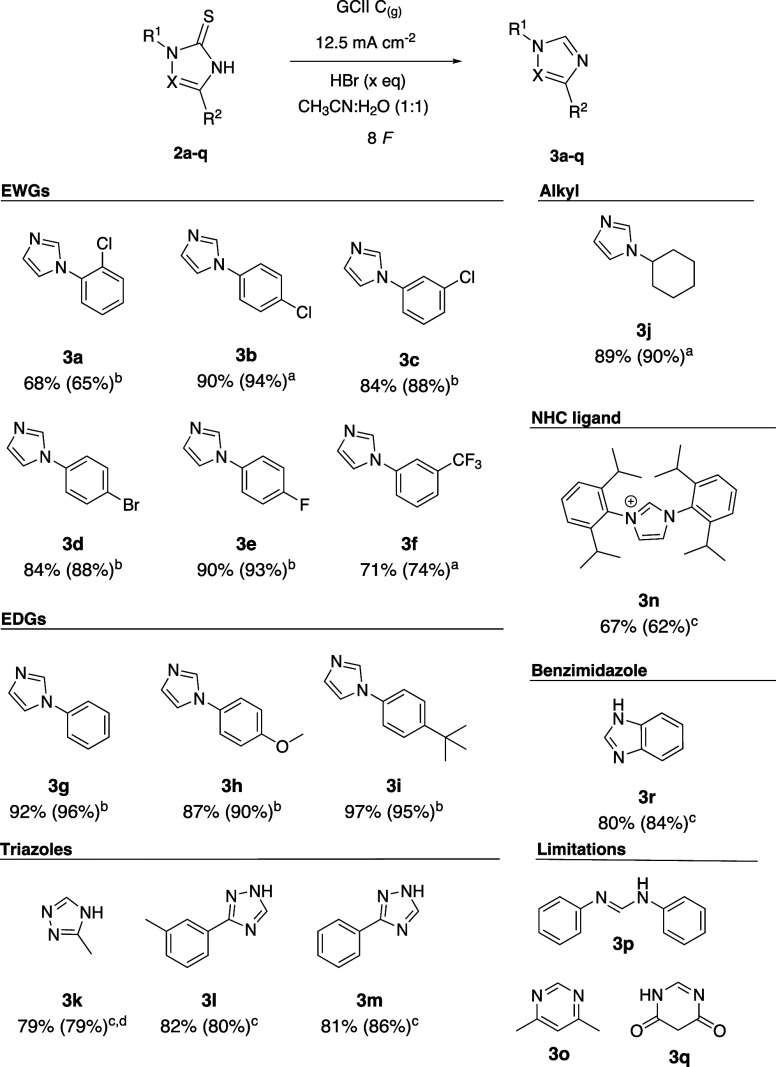
Scope of the anodic desulfurization to give the corresponding azole in isolated yield. Between parentheses is reported the ^1^H NMR yield using 1,3,5-trimethoxybenzene as internal standard: ^a^with HBr 0.5 equiv, ^b^10 equiv, ^c^1 equiv, and ^d^0.42 mmol of starting material instead of 0.14 mmol.

The study explored scaling up the electrochemical desulfurization of bis N-substituted imidazole **3n**, relevant as NHC ligand precursors in industrial processes (Figure 2).^37^ We chose to scale-up compound **2n** after a brief optimization (see SI, Table S3), We found some critical parameters: the solvent ratio, the equivalents of HBr and the shape of the stirring bar. Increasing the amount of acetonitrile enhances the solubility of **2n** in the media, while the shape of the stirring bar helps to drag down the floating and remaining insoluble starting material. Interestingly, the dependence on the equivalents of HBr was maintained: 0.5 equiv yielded 50% of the product, recovering 50% of the starting material. Using 1 equiv of HBr, we were able to reach 95% yield. This led us to suspect a correlation between counteranion availability and yield. This was proven when another counteranion was introduced in the system and the amount of HBr was lowered. The bromide is “free” from the imidazolinium and can continue the catalytic cycle. This could clarify why different substrates have different optimal conditions, since in the strong acidic media our products are protonated, and they are present in solution as salts. After the optimization of **3n**, a successful scale-up to 5 g was done to prove the reliability of the system with quantitative yields.

**Figure 2:**
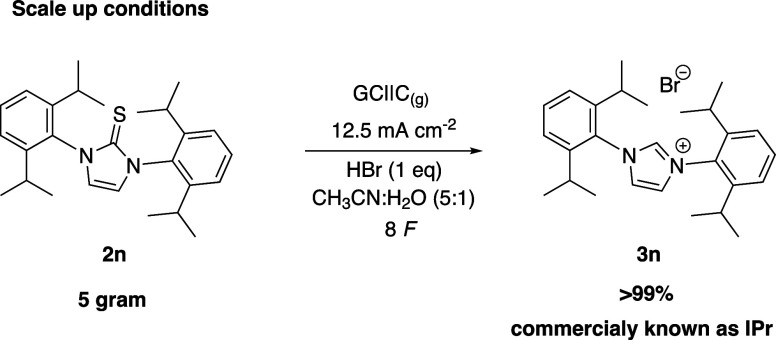
Scale-up of the anodic desulfurization to give an NHC ligand precursor.

Mechanistic experiments were used to clarify the mechanism (see SI). Adding a saturated solution of BaCO_3_ at the end of the electrolysis turned the solution into a milky one; this confirms the presence of sulfate derived from the oxidation of thione and in agreement with the literature.^13^ The cyclovoltammetry studies on molecule **2b** showed that an oxidation peak of the substrate led us to suspect a dimerization of the starting material as competitive to the anodic oxidation of the bromide. The dimerization for oxidative desulfurization is also reported in the literature.^13^

In summary, we established a novel anodic desulfurization that granted access to 14 diverse relevant heterocycles in good yield (up to 97% yield). The scope demonstrated the tolerance of the system to different scaffolds and substituents. This simple to conduct electrolysis employs undivided cells, constant current and carbon electrodes and readily available bromide as a mediator. The successful scale-up to 5 g of an industrially used NHC ligand showed a mature system for technical applications. Further studies about this reactivity could open new and greener ways to the synthesis of related scaffolds.

## Data Availability

The data underlying this study are available in the published article and its Supporting Information.

## Supporting Information Available

Experimental details, mechanistic studies and spectra of isolated compounds. (PDF)
