# Supplementary material for: Anodic Desulfurization of Heterocyclic Thiones – A Synthesis to Imidazoles and Analogues
Source: Org Lett. 2024 Oct 28;26(44):9476–80. doi: 10.1021/acs.orglett.4c03413 (PMC11555668; doi:10.1021/acs.orglett.4c03413)
Supplement: Supplementary file 1 — ol4c03413_si_001.pdf [file ol4c03413_si_001.pdf]

Supporting Information

Anodic Desulfurization of Heterocyclic Thiones – A Synthesis to Imidazoles and Analogues.

Davide Cesca, [a] Philip Arnold, [a] Dainis Kaldre, [b] Fabio Falivene, [c] Filippo Sladojevich, [c] Kurt Puentener, [b] Siegfried R. Waldvogel [a, d, f]

[a] Department of Chemistry, Johannes Gutenberg University, Duesbergweg 10–14, 55128 Mainz, Germany

[b] Department of Process Chemistry & Catalysis, F. Hoffmann-La Roche Ltd, 4070 Basel, Switzerland

[c] Pharma Research and Early Development, Roche Innovation Center Basel, F. Hoffmann-La Roche Ltd, 4070 Basel, Switzerland

[d] Department of Electrosynthesis, Max-Planck-Institute for Chemical Energy Conversion, Stiftstrasse 34-36, 45470 Mülheim an der Ruhr, Germany

[f] Karlsruher Institut für Technologie, Kaiserstraße 12, 76131 Karlsruhe, Germany

Contents

1. Materials and Methods S3

2. Additional Experimental Results S5

3. Experimental Procedures S9

   3.2 Synthesis of *N*,*N*-bis(2,6-diisopropylphenyl)ethane-1,2-diimine (**1n**)........ S9

   3.3 Synthesis of 1,3-bis(2,6-diisopropylphenyl)-imidazole -2-thione (**2n**) S10

   3.4 Synthesis of benzimidazole-2-thione (**2r**) S10

   3.5 Screening of the electrochemical oxidative desulfurization of 1-(4-chlorophenyl) imidazole-2-thione (**2b**) ... S10

   3.6 General procedure for the synthesis of the thioimidazoles 3a-3n derivatives........ S11

   3.7 Scale up of the electrochemical oxidation of the 1,3-bis(2,6-diisopropylphenyl)-imidazolium (**3n**) S12

4. Experimental Setup........ S13

5. Mechanicistic studies S14

6. Characterization of Compounds S17

NMR Spectra S26

References S54

1. Materials and Methods

The chemicals used were purchased commercially and were deployed without further purification, if not stated differently. Cyclohexane and ethyl acetate were bought with technical quality and were used after purification by distillation. If not stated differently, the reactions were carried out at room temperature.

Liquid Phase Chromatography

The preparative separations via normal phase „Flash chromatography" were performed on SILICA 60 M (0.040-0.063 mm), from the company *Macherey-Nagel GmbH &Co*, Düren, Germany. The separations without pressurization were performed with SILICA GEDURAN SI 60 (0.063-0.200 mm) from *Merck KGaA*, Darmstadt, Germany. Preparative column chromatography was also performed on prepacked puriFlash™ silica columns (15 µm or 30 µm, PF-30SIHP-F0012) using a puriFlash™-System (puriFlash™ XS520Plus) with an integrated UV detector from *Interchim*, Montlucon Cedex, France. Methanol (analytical reagent grade), ethyl acetate (distilled), cyclohexane (distilled)) and dichloromethane (analytical reagent grade) were used as eluents. The technical grade solvents were purified by distillation under reduced pressure, while dichloromethane and methanol were used without further purification.

The analytical separations using normal phase thin-layer chromatography (TLC) were performed with ALUMINUM TLC PLATES, SILICA GEL 60 F254 from *Merck KGaA*, Darmstadt, Germany. The R_f values are given according to the used solvent ratios.

The analytical measurements using high performance liquid chromatography – mass spectrometry (HPLC-MS) was carried out using a LCMS-2020, with the autosampler SIL-20AHT, a column oven CTO-20AC, two pump modules LC-20AD for the configuration of the eluent gradient, a photodiode array detector SPD-M20A, a HPLC modular system controller CBM-20A and a single quadrupole mass spectrometer from *Shimadzu*, Kyoto, Japan. The separations made use of a EUROSPHER II 100-5 C18 separation column from *Knauer*, Berlin, Germany. Acetonitrile (LC/MS grade) and a mixture of 5 % acetonitrile (LC/MS grade), 94.9 % water (ultrapure,type 1) and 0.1 % formic acid (98+%) were used as solvents.

Gas chromatography

The gas chromatographic (GC) analysis of product mixtures and pure substances was carried out using the GC-2030 gas chromatograph from S*himadzu*, Japan. It is used on a quartz capillary column HI-5MS from *Avantor* VWR, Radnor, USA (length: 30 m; inner diameter: 0.25 mm; film thickness of the covalently bonded stationary phase ((5 % phenyl)dimethylsiloxane): 0.25 µm; carrier gas: hydrogen at a constant velocity of 40 cm s-1. Injector temperature: 270 °C; detector temperature: 320 °C; program: "medium" method: 50 °C start temperature for 1 min, heating rate: 17.5 °C/min, 300 °C end temperature for 4.71 min). Gas chromatographic mass spectra: The analysis of the crude reaction mixtures and the purified products was performed using a GCMS-QP2010SE (*Shimadzu*, Kyoto, Japan) equipped with an electron ionization (EI) source and a quadrupole mass analyzer. A HI-5MS quartz capillary column (*Avantor* VWR, Radnor, USA) with the following specification was used: Length of 30 m, inner diameter of 0.25 mm and a stationary phase (5%-phenyl)dimethylsiloxane) of 0.25 µm thickness. Helium was used as the carrier gas at a constant velocity of 30 cm s^{-1}. The GC temperature ramp started at 50 °C (held for 1 min) and was heated up to 300 °C (held for 4.71 min) with a temperature ramp of 17.5 °C/min (method: 2_medium, total program duration: 20.0 min). The measurements were carried out at an injector temperature of 270 °C and an EI source temperature of 250 °C.

NMR Spectroscopy

Nuclear magnetic resonance measurements were performed using a multi core resonance spectrometer AVANCE III HD 300, AVANCE II 400 from *Bruker*, Analytische Messtechnik, Karlsruhe, Germany. Solid-state NMR were performed using a spectrometer Avance DSX 400 from *Bruker* Analytische Messtechnik, Karlsruhe, Germany, using 2.5 mm high range (100.0-163.0 MHz) and 4.0 mm high range (94.0-163.0 MHz) sample heads. $CDCl_3$ was used as the solvent. The ^1H and ^{13}C solution spectra were referenced in dependence of the residual signal of the non-deuterated solvent. The assignment of the ^1H and ^{13}C signals occurred partially with the help of COSY, HSQC and HMBC spectra. The chemical shifts are given as δ-values in ppm. Multiplets were labelled with following acronyms: s (singulet), t (triplet), q (quartet), m (multiplet), dd (doublet of doublet). All coupling constants *J* were listed with the amount of enclosed bonds in Hertz (Hz).

Cyclic Voltammetry (CV) Measurements

Cyclic voltammetry was performed using a PGSTAT204 potentiostat (Metrohm AG, Herisau, Switzerland) with a scan rate of 100 mV·s-1. Working electrode: GC electrode tip (0.3 mm diameter); counter electrode: glassy carbon rod; reference electrode: Ag/AgCl (vs. Fc^+/Fc = ferrocene; saturated LiCl in ethanol, Metrohm AG, Herisau, Switzerland). Prior to the CV measurements the electrolyte was purged with argon for 20–25 min. An argon atmosphere was kept flowing over the electrolyte during the measurements. The electrode potentials are reported with reference to the redox system ferrocene/ferrocenium (Fc^+/Fc)

2. Additional Experimental Results

Table S1 Screening of the conditions for the oxidative desulfurization of 1-(4-chlorophenyl) imidazole-2-thione (**2c**) with 0.5 equivalents of HBr

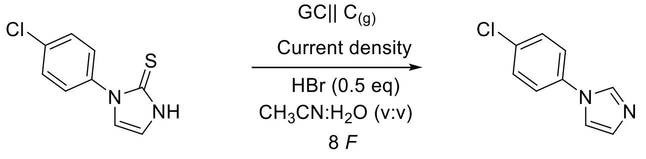

Entry	Concentration (M)	MeCN: H_2O (v:v)	Current density (mA cm^{-2})	Yield (%)[a]
1	0.028	50:50	6.25	72
2	0.028	50:50	9.38	84
3	0.028	50:50	12.50	94
4	0.028	50:50	18.75	71
5	0.028	50:50	25.00	73
6	0.028	50:50	37.50	74
7	0.028	70:30	12.5	81
8	0.028	30:70	12.5	63

a) Yield of **2a** was determined by 1H NMR spectroscopy using 1,3,5-trimethoxybenzene as internal standard.

Table S2 Screening of the conditions for the oxidative desulfurization of 1-(4-chlorophenyl) imidazole-2-thione (**2c**) with 10 equivalents of HBr

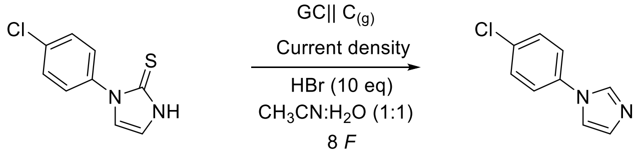

Entry	Concentration (M)	Current density (mA cm^{-2})	Yield (%)[a]
1	0.028	12.50	92
2	0.041	12.50	91
3	0.056	12.50	93

4	0.041	25.00	81
5	0.041	37.50	91

a) Yield of **2c** was determined by 1H NMR spectroscopy using 1,3,5-trimethoxybenzene as internal standard.

Table S3 Optimization of the conditions for the scale-up of the compound **3n**

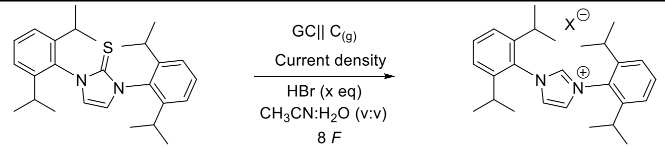

Entry	Solvent mixture	HBr (eq)	Additive	Yield (%)
1	1:1	0.5	-	50
2	1:1	1	-	83
3	5:1	1	-	95
4	5:1	0.5	$NaBF_4$ (1 eq)	98

a) Isolated yield.

Table S4 Comparison of the yields of **2a-2n** with different equivalents of HBr

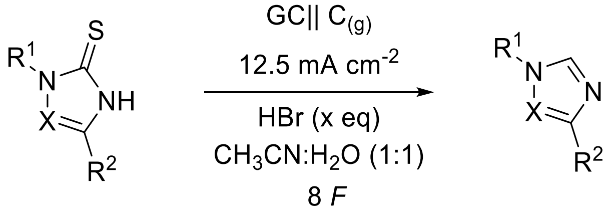

	Product	Yield with 0.5 eq of HBr [a]	Yield with 10 eq of HBr [a]	Yield with 1 eq of HBr [a]
3a	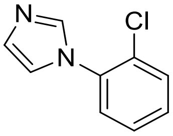	56%	65% (68%)	-
3b	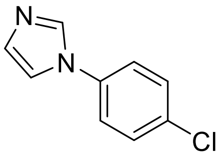	94% (90%)	92%	-
3c	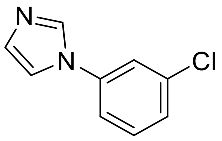	83%	88% (84%)	-
3d	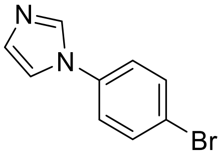	62%	88% (84%)	-
3e	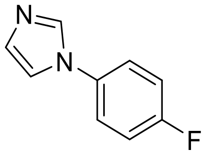	75%	93% (90%)	-
3f	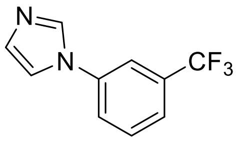	74% (71%)	75%	-
3g	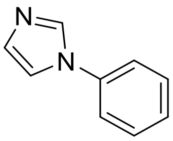	65%	96% (92%)	-
3h	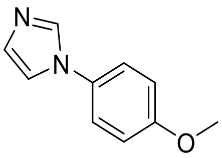	70%	90% (93%)	-

3i	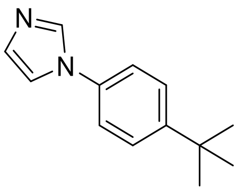	66%	95% (97%)	-
3j	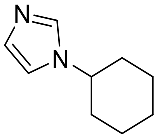	90% (89%)	71%	-
3k	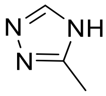	34%	53%	79% (79%)
3l	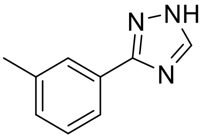	26%	89%	80% (82%)
3m	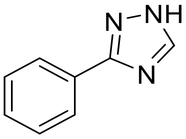	51%	79%	86% (81%)
3n	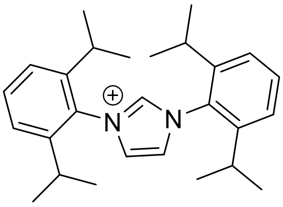	24%	67% (62%)	
3r	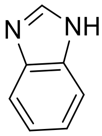	3%	84% (80%)	

a) Yield was determined by 1H NMR spectroscopy using 1,3,5-trimethoxybenzene as internal standard. In brackets are reported the isolated yield.

3. Experimental Procedures

3.1 General procedure for the synthesis of 1-substituted imidazole-2-thiones (2a-2j)

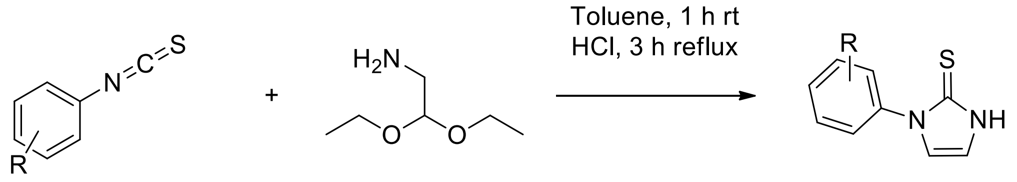

2a-j

A mixture of isothiocyanate (1.0 eq) and amino acetal (1.0 eq) in toluene was stirred at room temperature for 1 h. Concentrated HCl (37 wt. % in water, 0.5 eq) was added to the reaction mixture and was refluxed for 3 h. After that, the solvent was evaporated, and the residue was treated with water and sodium hydrogen carbonate (pH 8). The sediments were filtered and washed with hexane. The product was purified by recrystallization in ethanol or column chromatography.

Only the N-phenyl imidazole-2-thione and the 1(4-methoxyphenyl)-imidazole-2-thione were purchased.

3.2 Synthesis of *N,N*-bis(2,6-diisopropylphenyl)ethane-1,2-diimine (1n)

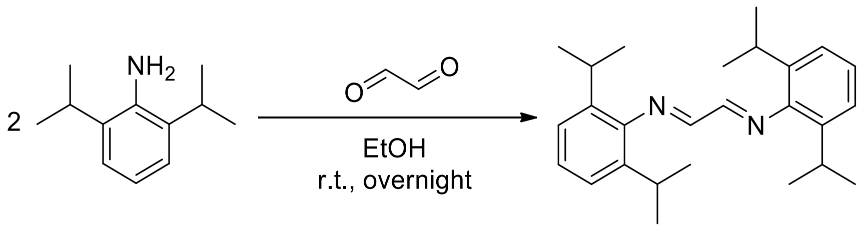

1n

N,N-bis(2,6-diisopropylphenyl)ethane-1,2-diimine (**1n**) was synthesized according to the procedure reported by Medvdko et.al. was used.[49] Accordingly, to a solution of 2,6 diisopropryl aniline (19.82 g, 112 mmol) in 150 mL of ethanol, 1.0 equivalents of glyoxal (40 wt.%, 8.33 g, 56 mmol) of aqueous glyoxal solution was added in one portion and left to stir overnight. The next day, the yellow solution was concentrated in vacuo and the residue was recrystallized from ethanol (300 mL) to provide 1n as a bright yellow crystalline solid (20.00 g, yield 95%, HPLC-UV purity 99.9%).

^{1}H NMR (400 MHz, $CDCl_3$) δ (ppm) = 8.11 (s, 2H), 7.18 (q, J=5.5, 6H), 2.94 (h, J=6.8, 4H), 1.22 (d, J=6.9, 24H).

^{13}C NMR (101 MHz, $CDCl_3$) δ (ppm) = 163.25, 148.15, 136.86, 125.27, 123.33, 28.19, 23.63, 23.53.

HR-MS (ESI+): calculated. for $[C_{26}H_{36}N_2 + H]^+$ =377.2951, found=377.2942

3.3 Synthesis of 1,3-bis(2,6-diisopropylphenyl)-imidazole -2-thione (2n)

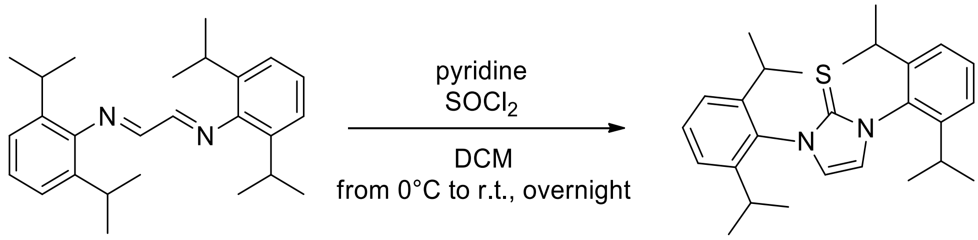

1n **2n**

To a solution of **1n** (20.0 g, 53.0 mmol) in 200 mL of DCM at 0°C, thiophosgene (6.09 g, 53.0 mmol) was added, subsequently of pyridine (16.75 g, 212.0 mmol) was added and left to stir overnight. The next day, the red/orange solution was washed with water and brine (10mL) and extracted three times (3x30mL) with dichloromethane. The combined organic phase was concentrated in vacuo and the residue was recrystallized from hot acetonitrile (100mL) to give 2n as a white crystalline solid (5.7 g, yield 40%, HPLC-UV purity 99.9%).

^{1}H NMR (400 MHz, $CDCl_3$) δ (ppm) = 7.50 – 7.41 (m, 2H), 7.29 (d, J=7.8, 4H), 6.84 (s, 2H), 2.75 (hept, J=6.9, 4H), 1.31 (d, J=6.9, 12H), 1.21 (d, J=6.9, 12H).

^{13}C NMR (101 MHz, $CDCl_3$) δ (ppm) = 146.60, 133.90, 130.19, 124.32, 119.09, 29.05, 24.32, 23.54.

HR-MS (ESI+): calculated. for $[C_{27}H_{36}N_2S + H]^+$ =421.2672, found=421.2666

3.4 Synthesis of benzimidazole-2-thione (2r)

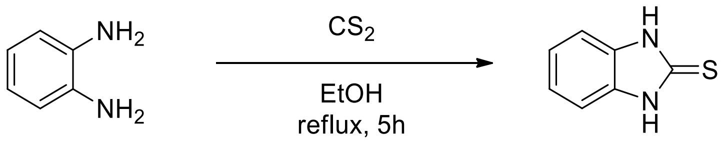

2r

The synthesize of the compound 2r was done according to the synthesis reported in literature.[50] To a solution of 1,2-benzenediamine (2.0 g, 18.5 mmol) in 20 mL of EtOH at r.t, carbon disulfide(4 mL, 66.5 mmol) was added. The solution was heated up to reflux and stirred for 5h. After letting the solution cool down to room temperature, the precipitate obtained was filtrated and washed 3 times with ethanol to give 2r as a white crystalline solid (1.42 g, yield 53%, HPLC-UV purity 99.9%).

^{1}H NMR (400 MHz, $CDCl_3$**)** δ (ppm) =10.17 (s, 2H) 7.28-7.09 (m, 6H)

^{13}C NMR (101 MHz, $CDCl_3$) δ (ppm) =

HR-MS (ESI+): calculated. for [$C_9H_8N_2$ + H]$^+$ =150.0252, found=

3.5 Screening of the electrochemical oxidative desulfurization of 1-(4-chlorophenyl) imidazole-2-thione (2b)

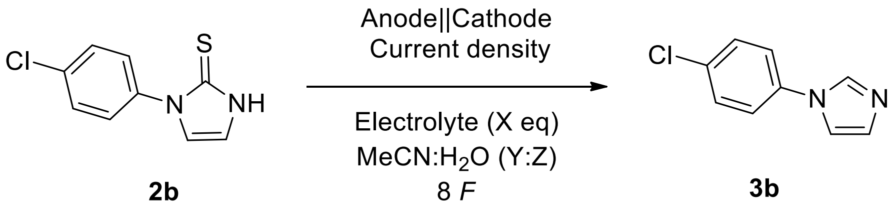

1-(4-chlorophenyl) imidazole-2-thione (29.4 mg, 0.14 mmol) was added to the corresponding solvent mixture of acetonitrile and water with the according supporting electrolyte in an undivided Teflon® cell (5 ml). The electrolysis (electrodes distance 1 cm) was then carried out at room temperature with 8 *F* and a stirring speed of 300 rpm. After the full conversion of the starting material, the aqueous layer was alkalized to pH 10, a saturated sodium chloride solution was added and was extracted 3 times (3x30mL) with dichloromethane. The combined extracts were dried over sodium sulfate and the solvent was distilled. Following, 1,3,5-trimethoxybenzene was added as an internal standard for quantifying the yield by ^{1}H NMR. The yield was then calculated by comparing selected signal integrals of the product with one of the internal standards.

3.6 General procedure for the synthesis of the thioimidazoles 3a-3n derivatives

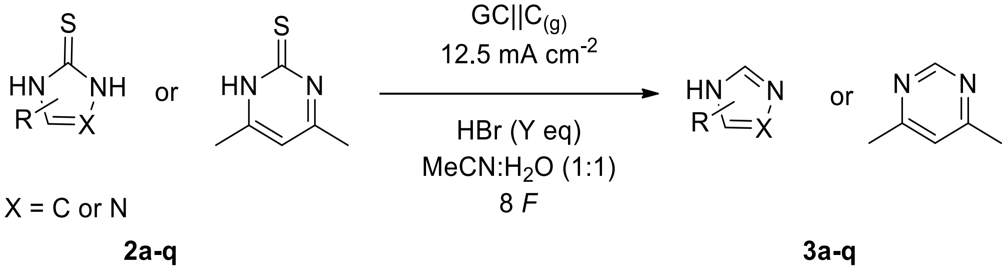

The imidazole-2-thione derivative **3a-q** (1.0 eq, 0.14 mmol) was dissolved in a mixture of water and acetonitrile (50:50) and HBr was added to the solution in a 5 mL Teflon® cell. The experiments were repeated with two different amounts of HBr: condition A with 0.5 eq, condition B with 10 eq. The electrolysis was carried out at room temperature with glassy carbon as anode and graphite as cathode material (electrodes distance 1 cm). The current density was set to 12.5 m*A* cm^{-2} and the stirring speed to 300 rpm. After 8 *F*, the reaction was stopped and worked up by adding a saturated sodium chloride solution and sodium hydrogen carbonate (pH 8). The substituted imidazoles were extracted 3 times (3x30mL) with dichloromethane and the triazoles 3 times (3x30mL) with ethyl acetate. The extracts were dried over sodium sulfate and the solvents were evaporated. The ^{1}H NMR yield was determined according to the example in GP2. The isolated yields were obtained after a chromatography column using ethyl acetate and cyclohexane as eluent.

3.7 Scale up of the electrochemical oxidation of the 1,3-bis(2,6-diisopropylphenyl)-imidazolium (3n)

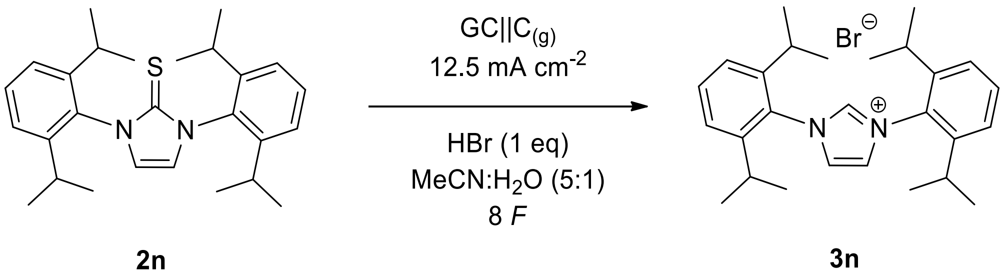

1,3-Bis(2,6-diisopropylphenyl)-imidazole -2-thione (5.00g, 12 mmol) was added to the corresponding solvent mixture of acetonitrile and water (300 mL) with the HBr (1 eq) in an glass cell (1 L). The electrolysis (electrodes distance 1 cm) was then carried out at room temperature with 8 *F* and a stirring speed of 500 rpm. After the full conversion of the starting material, the aqueous layer was neutralized with a saturated solution of sodium carbonate, a saturated sodium chloride solution was added and was extracted 3 times (3x30mL) with dichloromethane. The combined extracts were dried over sodium sulfate and the solvent was distilled. The solid obtained was dissolved in the minimum amount of ethyl acetate, the solution was then pass it through a silica plug (4 cm) and flashed 3 times with ethyl acetate (3x 50mL). The silica plug was then flashed with 100mL of methanol. The methanol solution was then placed in the rotavap to recover the product as colorless solid (5.61g, quantitative yield)

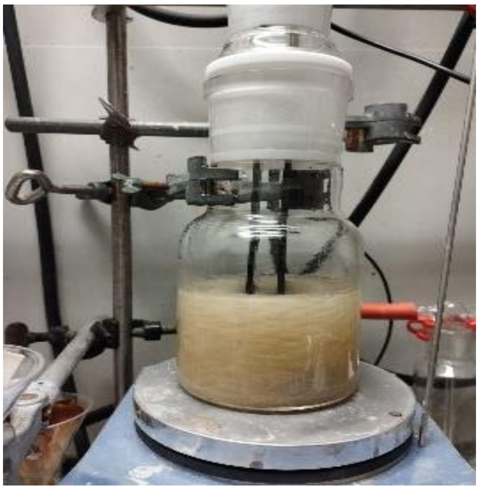

4. Experimental Setup

Electrochemical screening setup

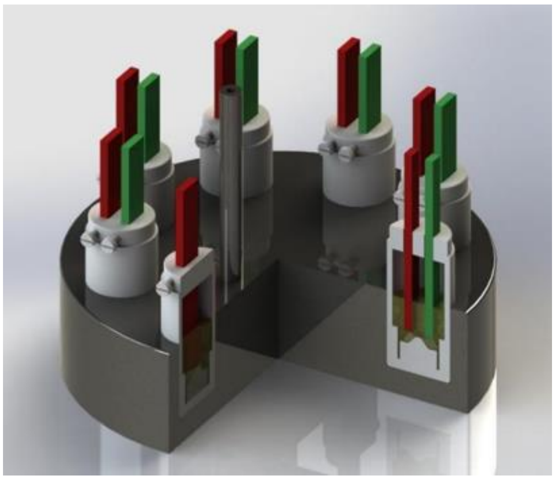

FigureS1: *Screening setup*

A Multichannel galvanostat HMP4040 (*Rohde & Schwarz*, Munich, Germany) was used in combination with undivided 5 mL Teflon® cells (*IKA® Werke GmbH & Co. KG,* Staufen, Germany) in a stainless-steel screening block (FigureS1: Screening *setup*). The cleaning of the stainless steel and graphite electrodes were performed with sandpaper (P600 and P1000) before use and then sonicated in a cyclohexene, water, and acetone emulsion. Glassy carbon electrodes were polished with alumina paste 1.0 μm. The used electrodes are listed in Table S5 with their producers.

Table S5: Electrode materials.

Entry	Electrode material	Producer
1	Isostatic graphite	*SGL Carbon*, Bonn, Germany
2	Glassy carbon (Sigradur®)	*HTW,* Thierhaupten, Germany
3	Sigraflex®	*SGL Carbon*, Meitingen, Germany
4	Stainless steel (VA 1.4571)	*Montanstahl GmbH,* Oelde, Germany

5. Mechanistic studies

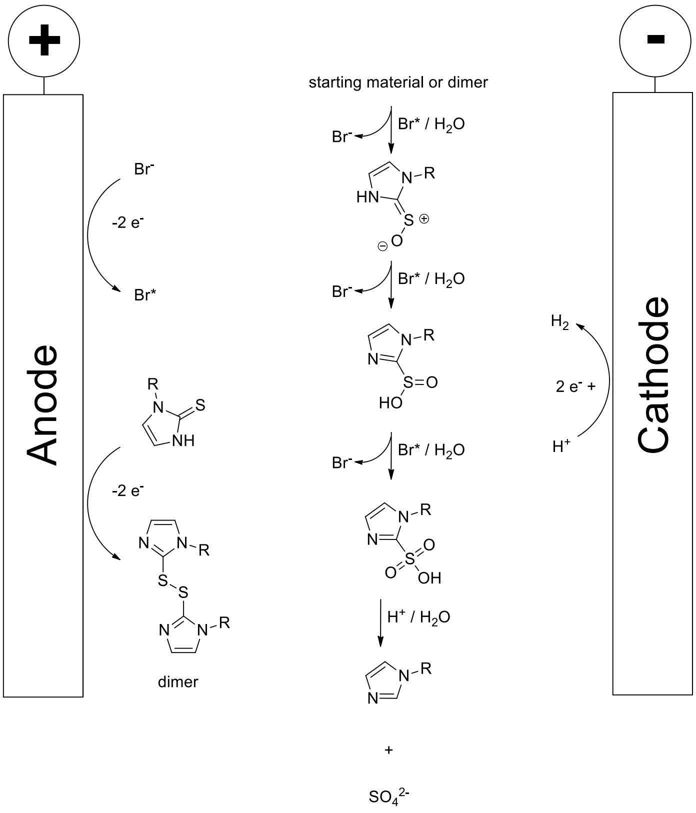

FigureS2: *Proposed mechanism*

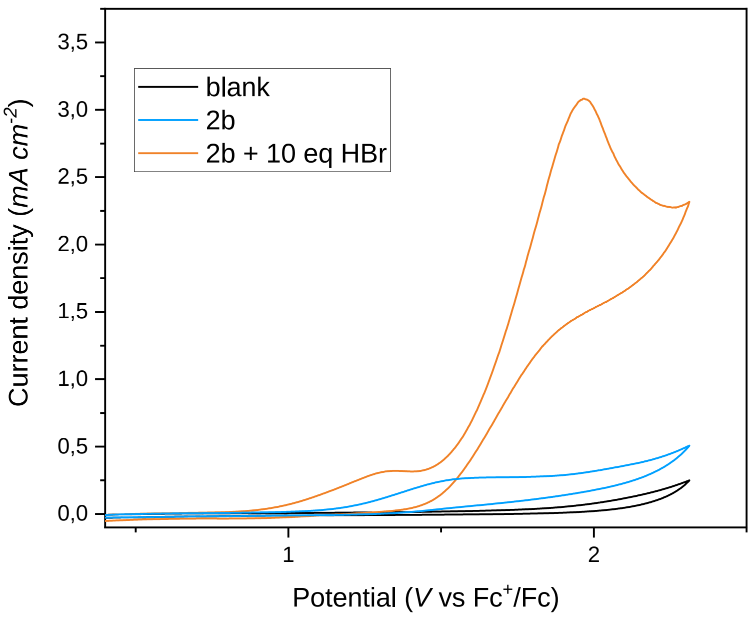

FigureS3: *CV of 10mM of 1-(4-chlorophenyl) imidazole-2-thione (2b) in a mixture of MeCN and water (1:1) with 0.1M TEABF*$_4$

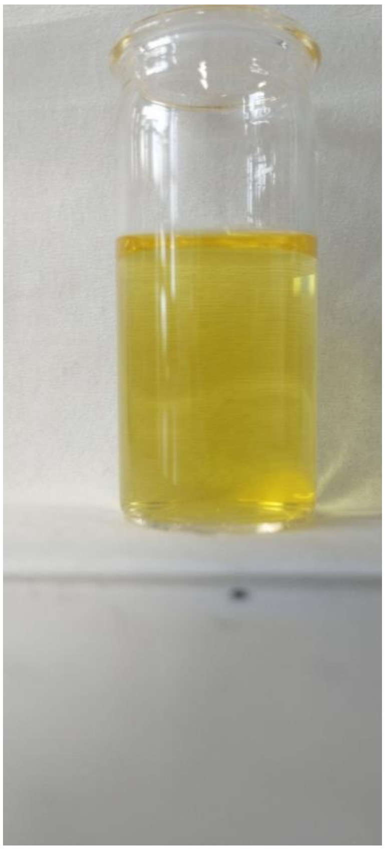

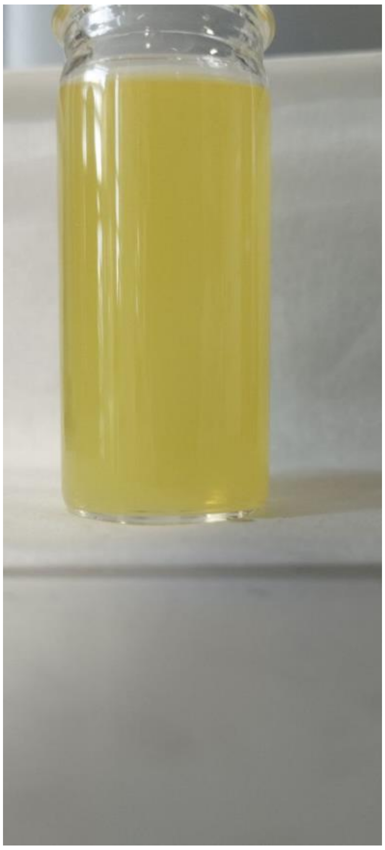

FigureS4: *Comparison of the solution after electrolysis. On the left the solution before adding a saturated solution of* $BaCO_3$*. On the right the solution after the addition, the milky solution confirms the presence of sulfate ions.*

6. Characterization of Compounds

1-(2-Chlorophenyl) imidazole-2-thione (**2a**)

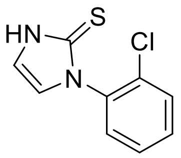

The desired product (**2a**) was isolated as colorless crystals.

(EtOAc:cyclohexane=20:80 -> 90: 10) Isolated 70%, 87.4 mg

^{1}H NMR (400 MHz, $CDCl_3$**)** δ (ppm) = δ 7.62 – 7.52 (m, 2H), 7.50 – 7.38 (m, 2H), 6.88 (d, J = 2.4 Hz, 1H), 6.82 (d, J = 2.4 Hz, 1H)

^{13}C NMR (101 MHz, $CDCl_3$) δ (ppm) = 134.9, 131.9, 130.8, 130.7, 130.2, 127.8, 120.0, 115.2

HR-MS (ESI+): calculated. for $[C_9H_7{}^{35}ClN_2S + H]^+$ =211.0091, found=211.0089

1-(4-Chlorohenyl) imidazole-2-thione (**2b**)

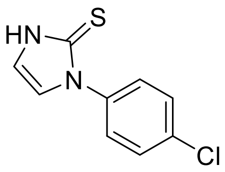

The desired product (**2c**) was isolated as colorless crystals. Spectroscopic matches those reported in literature. [51]

(Recrystallization from ethanol) Isolated 85%, 1.05g

^{1}H NMR (400 MHz, $CDCl_3$) δ (ppm) = 7.57 (d, 2H), 7.47 (d, 2H), 6.86 (dd, 2H).

^{13}C NMR (101 MHz, $CDCl_3$) δ (ppm) = 135.9, 134.5, 129.4, 127.3, 119.4, 115.1.

HR-MS (ESI+): calculated. for $[C_9H_7{}^{35}Cl\ N_2S + H]^+$ =211.0091, found=211.0090

1-(3-Chlorohenyl) imidazole-2-thione (**2c**)

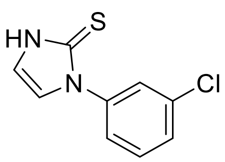

The desired product (**2b**) was isolated as colorless crystals. Spectroscopic matches those reported in literature. [51]

(EtOAc:cyclohexane=20:80 -> 90: 10) Isolated 72%, 93.9 mg

^{1}H NMR (400 MHz, $CDCl_3$**)** δ (ppm) = 7.67 (m, 1H), 7.58 (m, 1H), 7.51 – 7.41 (m, 2H), 6.89 (d, 1H), 6.87 (d, 1H).

^{13}C NMR (101 MHz, $CDCl_3$) δ (ppm) = 138.3, 134.8, 130.2, 128.9, 126.2, 124.3, 119.5, 115.4.

HR-MS (ESI+): calculated. for $[C_9H_7{}^{35}Cl\ N_2S + H]^+$ =211.0091, found=211.0093

1-(4-Bromophenyl) imidazole-2-thione (**2d**)

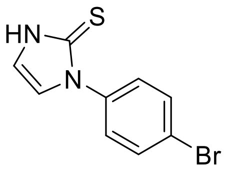

The desired product (**2d**) was isolated as brown powder.

(EtOAc:cyclohexane=20:80 -> 90: 10) Isolated 80%, 150.5 mg

^{1}H NMR (400 MHz, $CDCl_3$) δ (ppm) = 7.70 – 7.61 (m, 2H), 7.58 – 7.49 (m, 2H), 6.87(d, 2H), 6.83 (d, 2H).

^{13}C NMR (101 MHz, $CDCl_3$) □ (ppm) = 136.5, 132.4, 127.5, 122.5, 119.2, 114.9

HR-MS (ESI+): calculated. for $[C_9H_7{}^{79}BrN_2S + H]^+$ =254.9593, found=254.9601

1-(4-Fluorophenyl) imidazole-2-thione (**2e**)

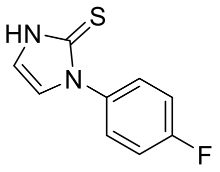

The desired product (**2e**) was isolated as colorless crystals.

(EtOAc:cyclohexane=20:80 -> 90: 10) Isolated 76%, 105.8 mg

^{1}H NMR (400 MHz, $CDCl_3$**)** δ (ppm) = 7.61 – 7.51 (m, 2H), 7.24 – 7.10 (m, 2H), 6.84 (q, J=2.4, 2H).

^{13}C NMR (101 MHz, $CDCl_3$) δ (ppm) = 163.6, 161.1, 133.6, 128.1, 119.7, 116.4

^{19}F NMR (376 MHz, $CDCl_3$) δ (ppm)= -111.95

HR-MS (ESI+): calculated. for $[C_9H_7FN_2S + H]^+$ =196.0414, found=196.0411

1-(3-(Trifluoromethyl)-phenyl) imidazole-2-thione (**2f**)

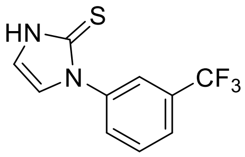

The desired product (**2f**) was isolated as colorless crystals. Spectroscopic matches those reported in literature. [51]

(EtOAc:cyclohexane=20:80 -> 90: 10) Isolated 88%, 159.7 mg

^{1}H NMR (400 MHz, $CDCl_3$**)** δ (ppm) = 7.94 – 7.86 (m, 2H), 7.67 (m, 2H), 6.91 (dd, J = 15.0, 2.9 Hz, 2H).

^{13}C NMR (101 MHz, $CDCl_3$) δ (ppm) = 161.4, 137.8, 131,9 131.3, 129.8, 129.5, 127.5, 125.4, 125.3, 124.8, 122.91, 122.87, 122.0, 119.2, 115.6

^{19}F NMR (376 MHz, $CDCl_3$) δ (ppm)= -62.77

HR-MS (ESI+): calculated. for $[C_{10}H_7F_3N_2S + H]^+$ =245.0355, found=245.0351

1-(4-Tert-butylphenyl) imidazole-2-thione (**2i**)

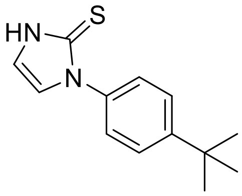

The desired product (**2i**) was isolated as colorless crystals.

(EtOAc:cyclohexane=0:100 -> 90: 10) Isolated 54%, 95.6 mg

^{1}H NMR (400 MHz, $CDCl_3$**)** δ (ppm) = 7.62 – 7.40 (m, 4H), 6.94 – 6.89 (m, 1H), 6.87 (q, J = 2.6 Hz, 1H), 1.35 (s, 12H)

^{13}C NMR (101 MHz, $CDCl_3$) δ (ppm) =151.8, 134.7, 126.2, 125.4, 120.14, 120.08, 115.2, 34.8, 31.3

HR-MS (ESI+): calculated. for $[C_{13}H_{16}N_2S + H]^+$ =233.1114, found=233.1129

1-Cyclohexyl imidazole-2-thione (**2j**)

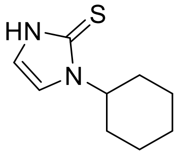

The desired product (**2j**) was isolated as colorless crystals. Spectroscopic matches those reported in literature. [51]

(EtOAc:cyclohexane=0:100 -> 90: 10) Isolated 45%, 50.3 mg

^{1}H NMR (400 MHz, $CDCl_3$) δ (ppm) = 6.77 (d, J = 2.4 Hz, 2H), 4.62 (tt, J = 11.6, 3.8 Hz, 1H), 2.09 (d, J = 3.6 Hz, 2H), 1.88 (dt, J = 13.5, 3.2 Hz, 2H), 1.81 – 1.67 (m, 1H), 1.58 – 1.33 (m, 4H), 1.29 – 1.12 (m, 1H)

^{13}C NMR (400 MHz, $CDCl_3$) δ (ppm) =158.2, 114.9, 114.7, 55.7, 32.6, 25.4, 25.3

HR-MS (ESI+): calculated. for $[C_9H_{14}N_2S + H]^+$ =183.0950, found=183.0948

Benzoimidazole (**2r**)

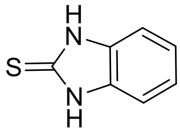

The desired product (**2r**) was isolated as colorless crystals. Spectroscopic matches those reported in literature. [50]

(washed with ethanol) Isolated 53%, 1.42 g

^{1}H NMR (400 MHz, $CDCl_3$**)** δ (ppm) =10.17 (s, 2H) 7.28-7.09 (m, 6H)

^{13}C NMR (101 MHz, $CDCl_3$) δ (ppm) = 170.8, 133.5, 123.4, 110.3

HR-MS (ESI+): calculated. for $[C_9H_8N_2 + H]^+$ =150.0252, found=150.0248

<u>1-(2-Chlorohenyl) imidazole (**3a**)</u>

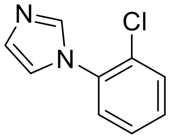

The desired product (**3a**) was isolated as colorless crystals. Spectroscopic matches those reported in literature. [52]

(EtOAc:cyclohexane=20:80 -> 100: 0) Isolated 68%, 16.9 mg

^{1}H NMR (400 MHz, $CDCl_3$**)** δ (ppm) = 7.73 (s, 1H), 7.56 (m, 1H), 7.45 – 7.32 (m, 3H), 7.22 (s, 1H), 7.18 (s, 1H).

^{13}C NMR (101 MHz, $CDCl_3$) δ (ppm) =137.5, 135.1, 130.9, 129.9, 129.8, 129.2, 127.9, 127.7, 120.5

HR-MS (ESI+): calculated. for $[C_9H_8{}^{35}Cl\ N_2 + H]^+$ =178.0298, found=178.0290

<u>1-(4-Chlorohenyl) imidazole (**3b**)</u>

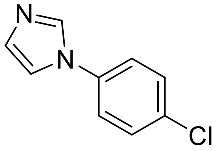

The desired product (**3c**) was isolated as colorless crystals. Spectroscopic matches those reported in literature. [54]

(EtOAc:cyclohexane=20:80 -> 100: 0) Isolated 84%, 20.9 mg

^{1}H NMR (400 MHz, $CDCl_3$) δ (ppm) = 7.81 (s, 1H), 7.51 – 7.43 (m, 2H), 7.40 – 7.33(m, 2H), 7.29 (s, 1H), 7.16 (s, 1H).

^{13}C NMR (101 MHz, $CDCl_3$) δ (ppm) = 136.5, 135.9, 133.2, 130.9, 130.3, 123.1, 118.5

HR-MS (ESI+): calculated. for $[C_9H_7{}^{35}Cl\ N_2 + H]^+$ =178.0298, found=178.0293

<u>1-(3-Chlorohenyl) imidazole (**3c**)</u>

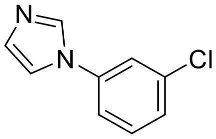

The desired product (**3b**) was isolated as colorless crystals. Spectroscopic matches those reported in literature. [53]

(EtOAc:cyclohexane=20:80 ->100: 0) Isolated 90%, 22.4 mg

^{1}H NMR (400 MHz, $CDCl_3$**)** δ (ppm) = δ = 7.71 (s, 1H), 7.54 (m, 1H), 7.42 – 7.30 (m, 3H), 7.20 (s, 1H), 7.15 (s, 1H)

^{13}C NMR (101 MHz, $CDCl_3$) δ (ppm) = 138.5, 135.6, 135.5, 130.9, 130.7, 127.7, 121.8, 119.5, 118.7

HR-MS (ESI+): calculated. for $[C_9H_8{}^{35}Cl\ N_2 + H]^+$ =178.0298, found=178.0296

1-(4-Bromophenyl) imidazole (**3d**)

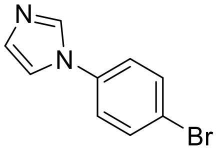

The desired product (**3d**) was isolated as colorless crystals. Spectroscopic matches those reported in literature. [55]

(EtOAc:cyclohexane=20:80 -> 100: 0) Isolated 84%, 26.2 mg

^{1}H NMR (400 MHz, $CDCl_3$) δ (ppm) = 7.94 (s, 1H), 7.66 – 7.57 (m, 2H), 7.32 – 7.27 (m, 2H), 7.28 – 7.22 (m, 2H)

^{13}C NMR (101 MHz, $CDCl_3$) δ (ppm) = 136.4, 135.6, 133.2, 130.4, 123.2, 121.3, 118.4.

HR-MS (ESI+): calculated. for $[C_9H_8{}^{79}BrN_2S + H]^+$ =222.9873, found=222.9882

1-(4-Fluorophenyl) imidazole (**3e**)

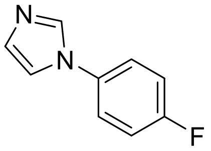

The desired product (**3e**) was isolated as colorless crystals. Spectroscopic matches those reported in literature. [54]

(EtOAc:cyclohexane=20:80 -> 100: 0) Isolated 90%, 20.5 mg

^{1}H NMR (400 MHz, $CDCl_3$**)** δ (ppm) = 7.82 (s, 1H), 7.46 – 7.27 (m, 2H), 7.25 – 7.07 (m, 4H).

^{13}C NMR (101 MHz, $CDCl_3$) δ (ppm) = 162.9, 160.5, 135.8, 133.58, 133.55, 130.2, 123.6, 123.5, 118.7, 116.9, 116.7.

^{19}F NMR (376 MHz, $CDCl_3$) δ (ppm)= -113.75

HR-MS (ESI+): calculated. for $[C_9H_8FN_2 + H]^+$ =163.0673, found=163.0671

1-(3-(Trifluoromethyl)-phenyl) imidazole (**3f**)

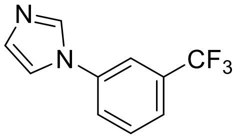

The desired product (**3f**) was isolated as colorless crystals. Spectroscopic matches those reported in literature. [56]

(EtOAc:cyclohexane=20:80 -> 100: 0) Isolated 71%, 21.1 mg

^{1}H NMR (400 MHz, $CDCl_3$**)** δ (ppm) =7.97 (s, 1H), 7.69 – 7.56 (m, 4H), 7.33 (s, 1H), 7.26 (s, 1H).

^{13}C NMR (101 MHz, $CDCl_3$) δ (ppm)= 137.9, 135.7, 133.3, 132.9, 132.6, 132.3, 130.9, 124.8, 124.5, 124.5, 124.5, 124.4, 122.1, 118.6, 118.6, 118.5, 118.5, 118.3.

^{19}F NMR (376 MHz, $CDCl_3$) δ (ppm)= -62.67

HR-MS (ESI+): calculated. for $[C_{10}H_8F_3N_2 + H]^+$ =213.0639, found=213.0637

1-Phenyl imidazole (**3g**)

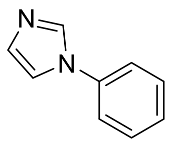

The desired product (**3g**) was isolated as colorless crystals. Spectroscopic matches those reported in literature. [55]

(EtOAc:cyclohexane=20:80 -> 100: 0) Isolated 92%, 18.7 mg

^{1}H NMR (400 MHz, $CDCl_3$**)** δ (ppm) = 7.90 (s, 1H), 7.56 – 7.42 (m, 2H), 7.42 – 7.33 (m, 3H) , 7.37 (s, 1H), 7.21 (s, 1H)

^{13}C NMR (101 MHz, $CDCl_3$) δ (ppm) = 137.3, 135.5, 129.9, 127.7, 121.6, 118.4

HR-MS (ESI+): calculated. for $[C_9H_8N_2 + H]^+$ =145.0765, found=145.0762

1-(4-Methoxyphenyl) imidazole (**3h**)

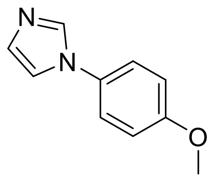

The desired product (**3h**) was isolated as colorless crystals. Spectroscopic matches those reported in literature. [55]

(EtOAc:cyclohexane=20:80 -> 100: 0) Isolated 93%, 22.7 mg

^{1}H NMR (400 MHz, $CDCl_3$**)** δ (ppm) = 7.77 (s, 1H), 7.38 – 7.23 (m, 2H), 7.16 (d, J=8.7, 2H), 6.96 (s, 1H), 6.94 (s, 1H), 3.81 (s, 3H).

^{13}C NMR (101 MHz, $CDCl_3$) δ (ppm) = 158.9, 135.8, 130.7, 129.9, 123.2, 118.8, 114.9, 55.6.

HR-MS (ESI+): calculated. for $[C_{10}H_{10}N_2O + H]^+$ =175.0871, found=175.0868

1-(4-Tert-butylphenyl) imidazole (**3i**)

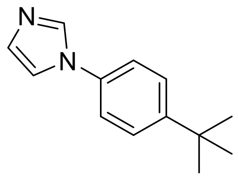

The desired product (**3i**) was isolated as colorless crystals. Spectroscopic matches those reported in literature. [57]

(EtOAc:cyclohexane=10:90 -> 100: 0) Isolated 97%, 27.3 mg

^{1}H NMR (400 MHz, $CDCl_3$**)** δ (ppm) = 7.87 (s, 1H), 7.55 – 7.45 (m, 2H), 7.38 – 7.29 (m, 2H), 7.27 (dd, J=3.3, 1.9, 1H), 7.21 (s, 1H), 1.36 (s, 9H).

^{13}C NMR (101 MHz, $CDCl_3$) δ (ppm) = 151.0, 135.7, 134.9, 130.0, 126.9, 121.4, 118.6, 34.8, 31.4, 2.1

HR-MS (ESI+): calculated. for $[C_{13}H_{16}N_2 + H]^+$ =201.1391, found=201.1388

<u>1-Cyclohexyl imidazole (**3j**)</u>

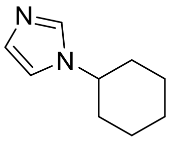

The desired product (**3j**) was isolated as colorless crystals.

(EtOAc:cyclohexane=10:90 -> 100: 0) Isolated 89%, 18.8 mg

^1H NMR (400 MHz, $CDCl_3$) δ (ppm) =7.55 (s, 1H), 7.02 (s, 1H), 6.94 (s, 1H), 3.92 (tt, J = 11.8, 3.8 Hz, 1H), 2.11 (ddq, J = 12.7, 4.0, 2.2 Hz, 2H), 1.91 (dt, J = 13.8, 3.5 Hz, 2H), 1.75 (dddt, J = 13.4, 5.2, 3.4, 1.7 Hz, 1H), 1.63 (qd, J = 12.4, 3.5 Hz, 2H), 1.52 – 1.17 (m, 3H).

^{13}C NMR (101 MHz, $CDCl_3$) δ (ppm) = 135.3, 128.7, 116.9, 56.8, 34.4, 33.5, 25.4, 25.2

HR-MS (ESI+): calculated. for $[C_9H_{14}N_2 + H]^+$ =151,2287, found=151.2290

<u>4*H* -3-Methyl-1,2,4-triazole (**3k**)</u>

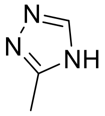

The desired product (**3k**) was isolated as colorless crystals. Spectroscopic matches those reported in literature. [57]

(EtOAc:cyclohexane=20:80 -> 100: 0) Isolated 79%, 48.4 mg

^1H NMR (400 MHz, $CDCl_3$) δ (ppm) = 8.09 (s, 1H), 2.56 (s, 3H).

^{13}C NMR (101 MHz, $CDCl_3$) δ (ppm) = 155.0, 148.5, 12.4.

HR-MS (ESI+): calculated. for $[C_3H_5N_3 + H]^+$ =84.0561, found=84.0560

<u>4*H* -3-(o-Tolyl)-1,2,4-triazole (**3l**)</u>

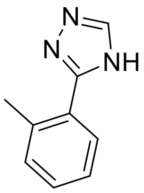

The desired product (**3l**) was isolated as colorless crystals.

(EtOAc:cyclohexane=20:80 -> 100: 0) Isolated 79%, 17.7 mg

^1H NMR (400 MHz, $CDCl_3$) δ (ppm) = 8.25 (s, 1H), 7.88 – 7.77 (m, 2H), 7.33 (t, J=7.6, 1H), 7.28 – 7.23 (m, 1H), 2.37 (s, 3H).

^{13}C NMR (101 MHz, $CDCl_3$) δ = 158.9, 147.7, 138.8, 131.0, 128.9, 128.3, 127.2, 123.6, 21.3.

HR-MS (ESI+): calculated. for $[C_9H_9N_3 + H]^+$ =160.0874, found=160.0876

4*H*-3-Phenyl-1,2,4-triazole (**3m**)

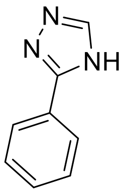

The desired product (**3m**) was isolated as colorless crystals.

(EtOAc:cyclohexane=20:80 -> 100: 0) Isolated 81%, 16.7 mg

^1H NMR (400 MHz, $CDCl_3$) δ = 8.29 (s, 1H), 8.10 – 7.99 (m, 2H), 7.52 – 7.39 (m, 3H)

^{13}C NMR (101 MHz, CDCl3) δ =159.1, 147.4, 130.2, 128.9, 128.6, 126.6.

HR-MS (ESI+): calculated. for $[C_8H_7N_3 + H]^+$ =146.0718, found=146.0717

N,N-Bis(2,6-diisopropylphenyl)ethane-1,2-diimine (**1n**)

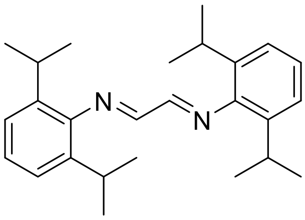

The desired product (**1n**) was isolated as yellow crystals. Spectroscopic matches those reported in literature. [49]

^1H NMR (400 MHz, $CDCl_3$) δ (ppm) = 8.11 (s, 2H), 7.18 (q, J=5.5, 6H), 2.94 (h, J=6.8, 4H), 1.22 (d, J=6.9, 24H).

^{13}C NMR (101 MHz, $CDCl_3$) δ (ppm) = 163.3, 148.3, 136.9, 125.3, 123.3, 28.2, 23.6, 23.5.

HR-MS (ESI+): calculated. for $[C_{26}H_{36}N_2 + H]^+$ =377.2951, found=377.2942

1,3-Bis(2,6-diisopropylphenyl)-imidazole -2-thione (**2n**)

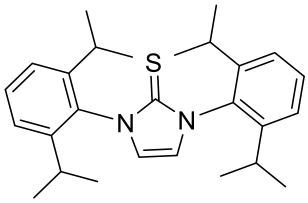

The desired product (**2n**) was isolated as colorless crystals. Spectroscopic matches those reported in literature. [49]

^1H NMR (400 MHz, $CDCl_3$) δ (ppm) = 7.50 – 7.41 (m, 2H), 7.29 (d, J=7.8, 4H), 6.84 (s, 2H), 2.75 (hept, J=6.9, 4H), 1.31 (d, J=6.9, 12H), 1.21 (d, J=6.9, 12H).

^{13}C NMR (101 MHz, $CDCl_3$) δ (ppm) = 146.6, 133.9, 130.2, 124.3, 119.1, 29.1, 24.3, 23.5.

HR-MS (ESI+): calculated. for $[C_{27}H_{36}N_2S + H]^+$ =421.2672, found=421.2666

1,3-bis(2,6-diisopropylphenyl)-imidazolium bromide(**3n**)

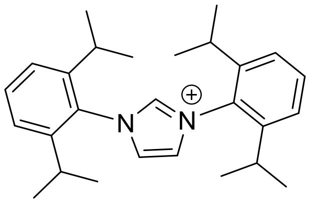

The desired product (**3n**) was isolated as colorless crystals. Spectroscopic matches those reported in literature. [49]

^{1}H NMR (400 MHz, $CDCl_3$) δ (ppm) = 9.97 (t, J=1.6, 1H), 8.12 (d, J=1.6, 2H), 7.57 (t, J=7.8, 2H), 7.34 (d, J=7.8, 4H), 2.43 (hept, J=6.9, 4H), 1.26 (dd, J=17.8, 6.8, 24H)

^{13}C NMR (101 MHz, $CDCl_3$) δ (ppm) = 145.1, 138.5, 132.3, 129.9, 126.9, 124.9, 29.3, 24.9, 23.9.

HR-MS (ESI+): calculated. for $[C_{27}H_{37}N_2]^+$ =389.2951, found=389.2954

Benzoimidazole (**3r**)

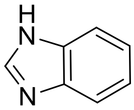

The desired product (**3r**) was isolated as colorless crystals. Spectroscopic matches those reported in literature. [58]

(EtOAc:cyclohexane=20:80 -> 100: 0) Isolated 80%, 13.2 mg

^{1}H NMR (400 MHz, $CDCl_3$) δ (ppm) = 8.11 (s, 1H), 7.71– 7.63 (m, 2H), 7.34 – 7.27 (m, 2H)

^{13}C NMR (101 MHz, $CDCl_3$) δ (ppm) = 140.5, 137.6, 123.5, 115.6

HR-MS (ESI+): calculated. for $[C_7H_7N_2 + H]^+$ =120.0682, found=120.0686

NMR Spectra

400 MHz, $CDCl_3$

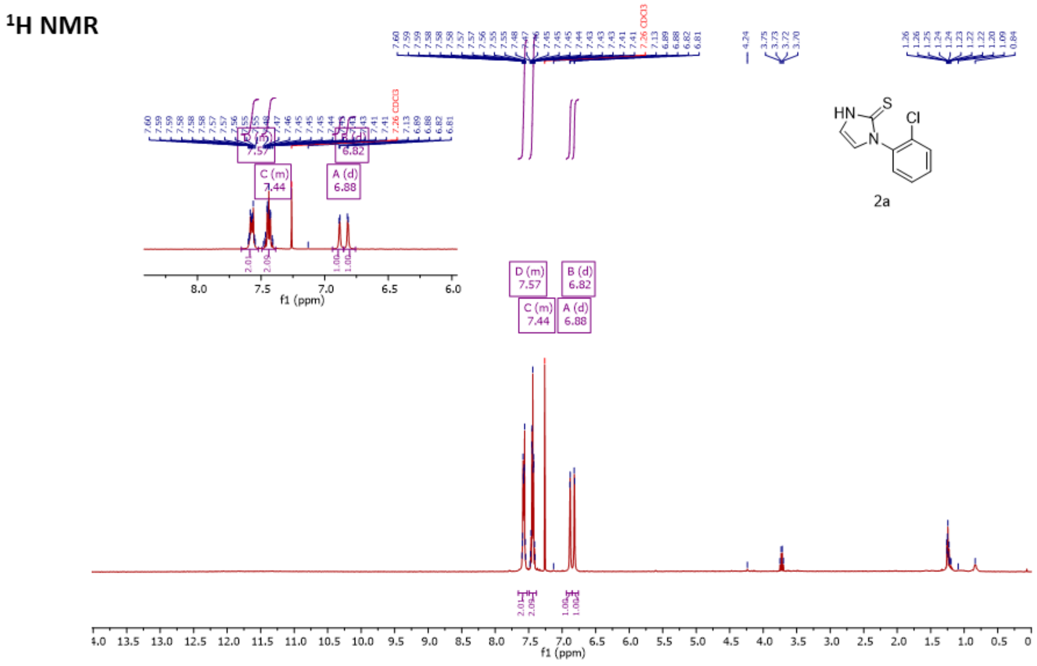

101 MHz, $CDCl_3$

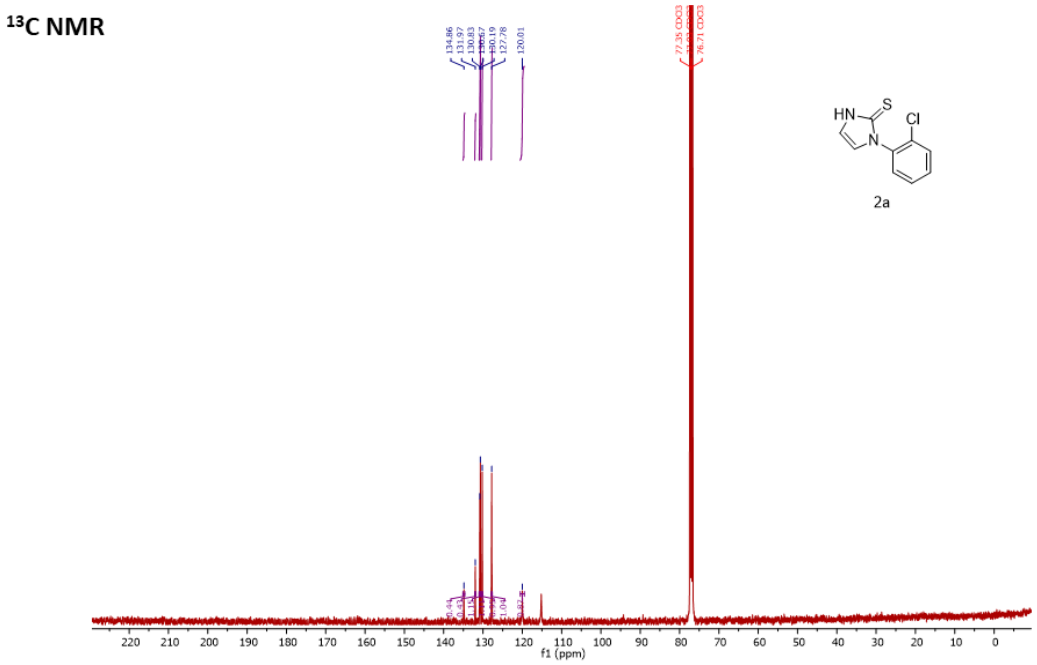

400 MHz, $CDCl_3$

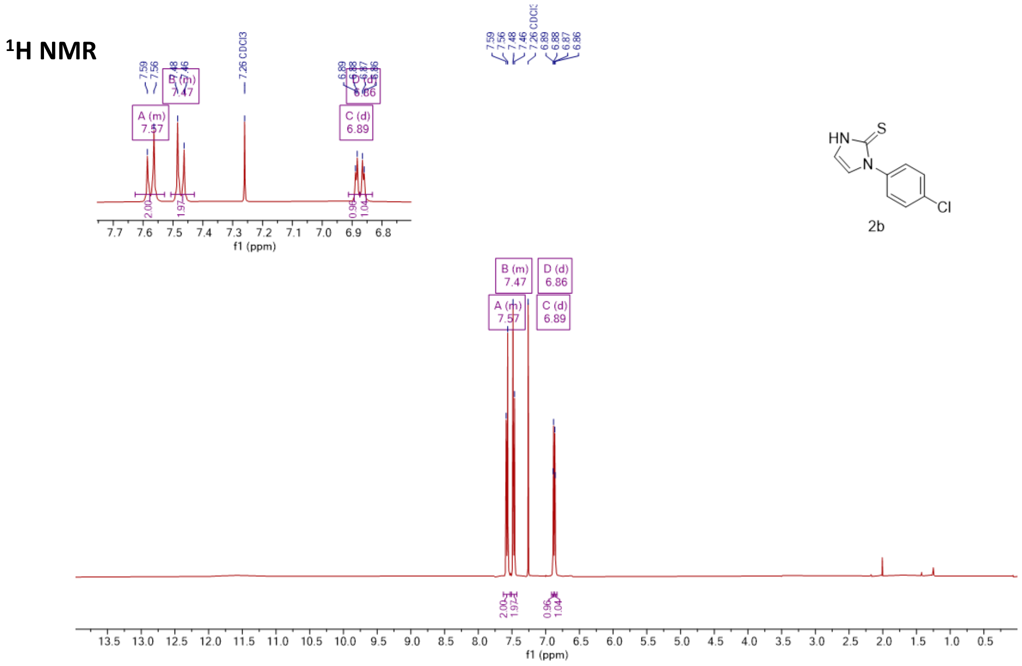

101 MHz, $CDCl_3$

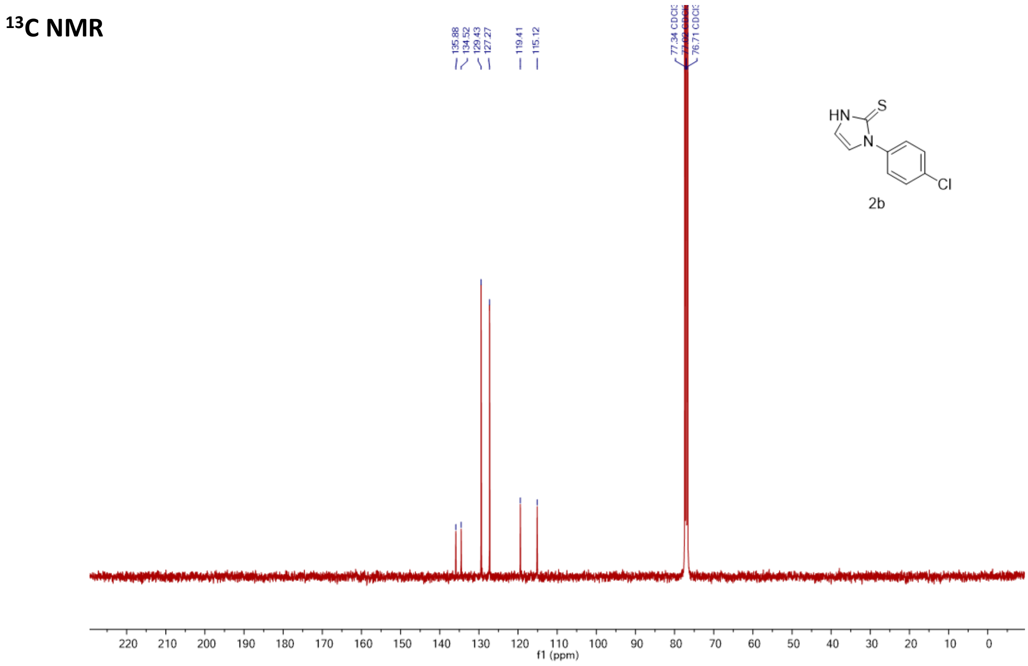

400 MHz, $CDCl_3$

^{1}H NMR

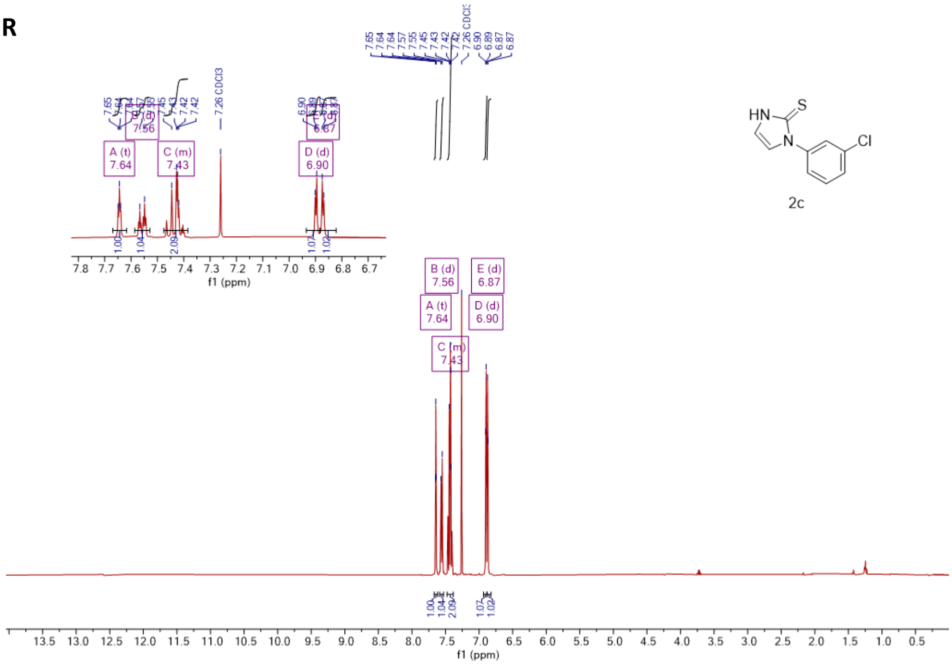

101 MHz, $CDCl_3$

^{13}C NMR

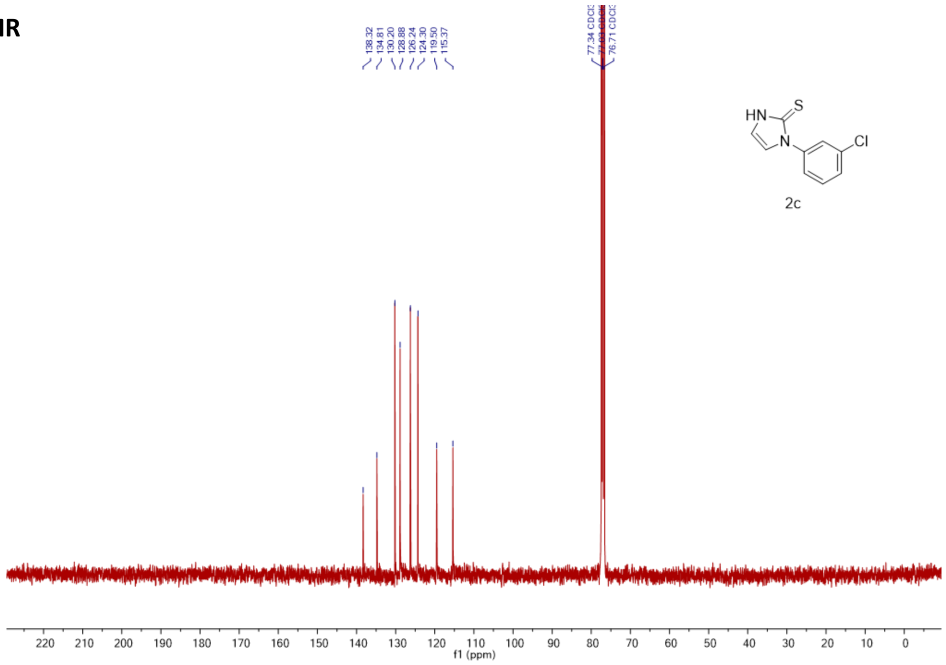

400 MHz, $CDCl_3$

^{1}H NMR

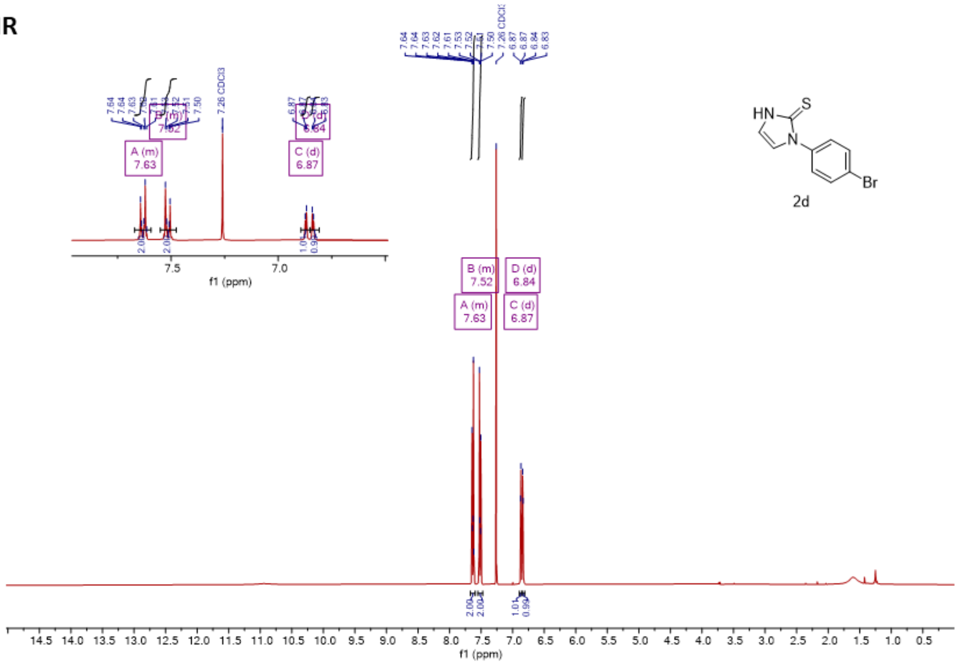

101 MHz, $CDCl_3$

^{13}C NMR

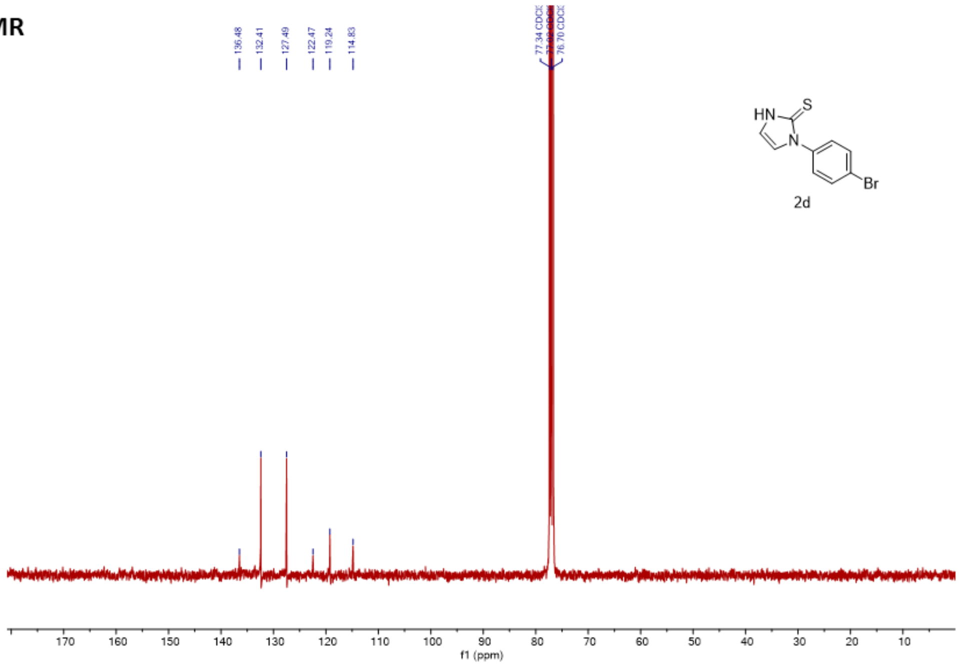

400 MHz, $CDCl_3$

^{1}H NMR

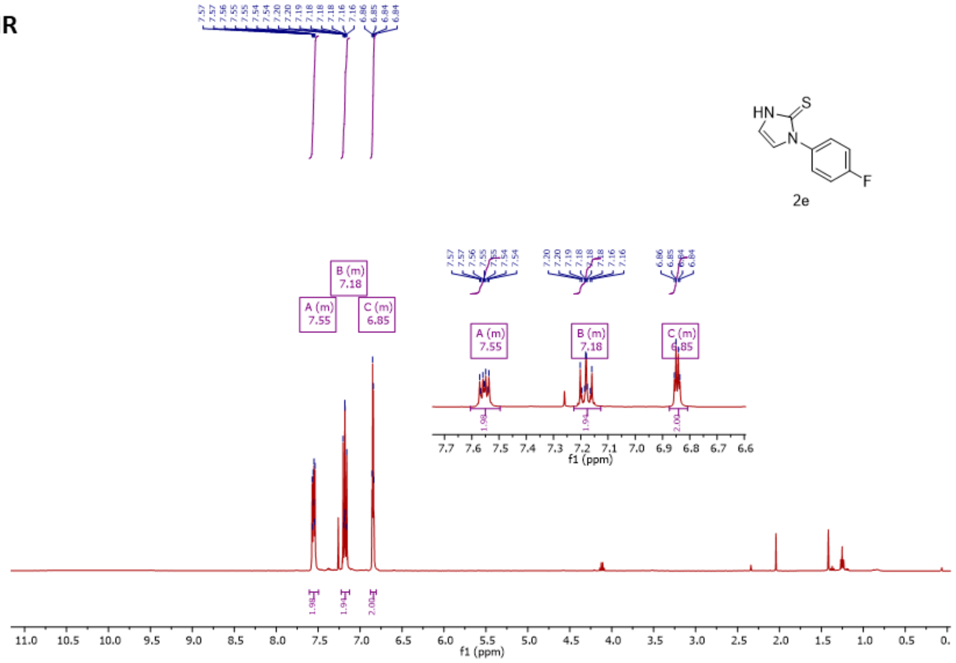

101 MHz, $CDCl_3$

^{13}C NMR

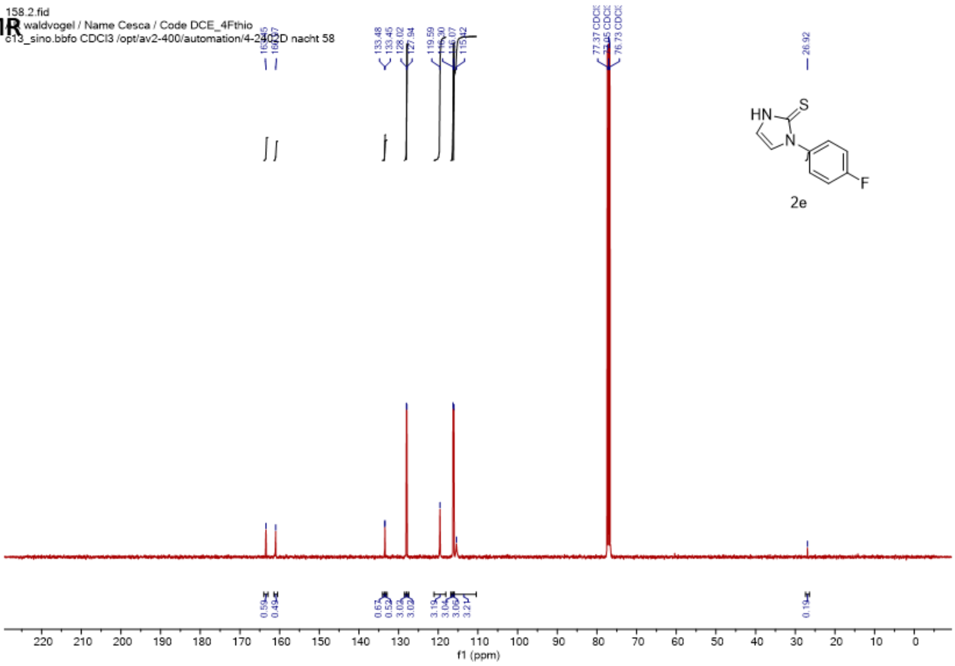

376 MHz, $CDCl_3$

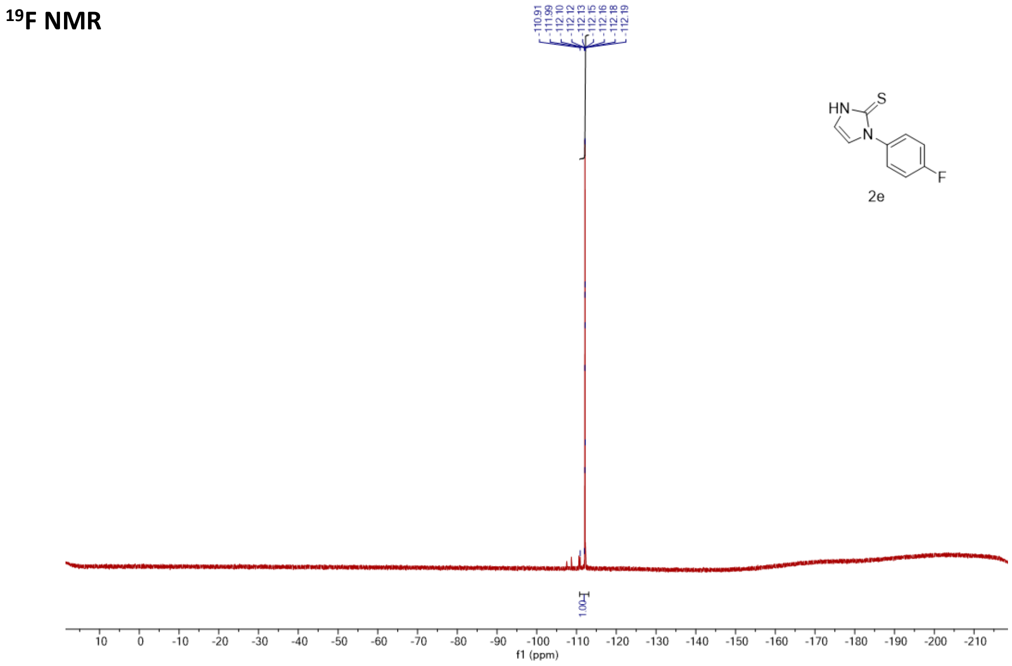

400 MHz, $CDCl_3$

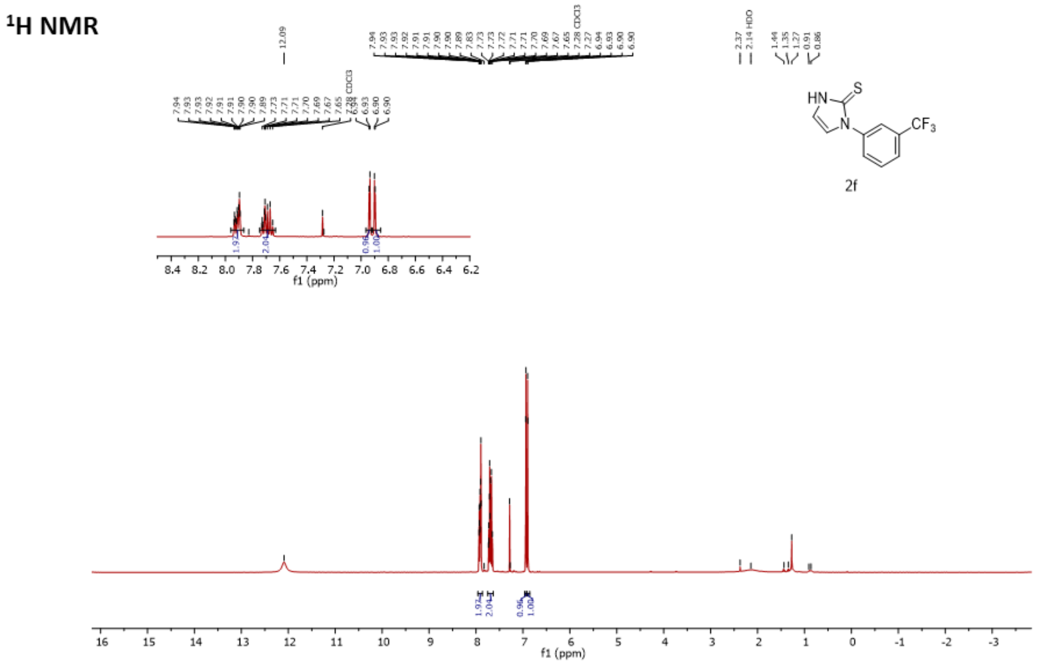

101 MHz, $CDCl_3$

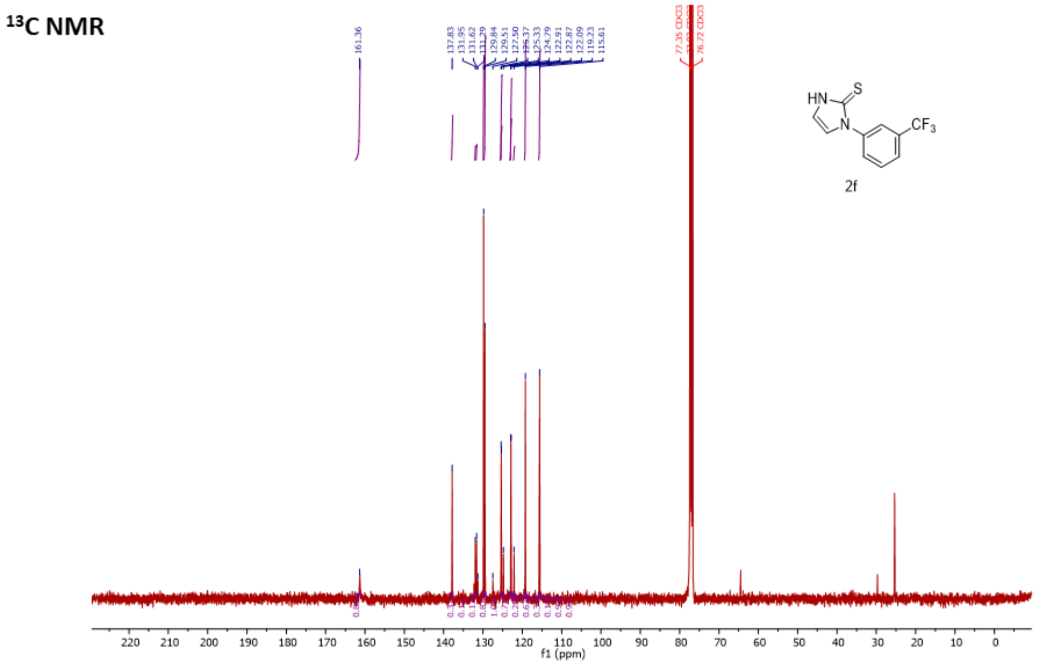

376 MHz, $CDCl_3$

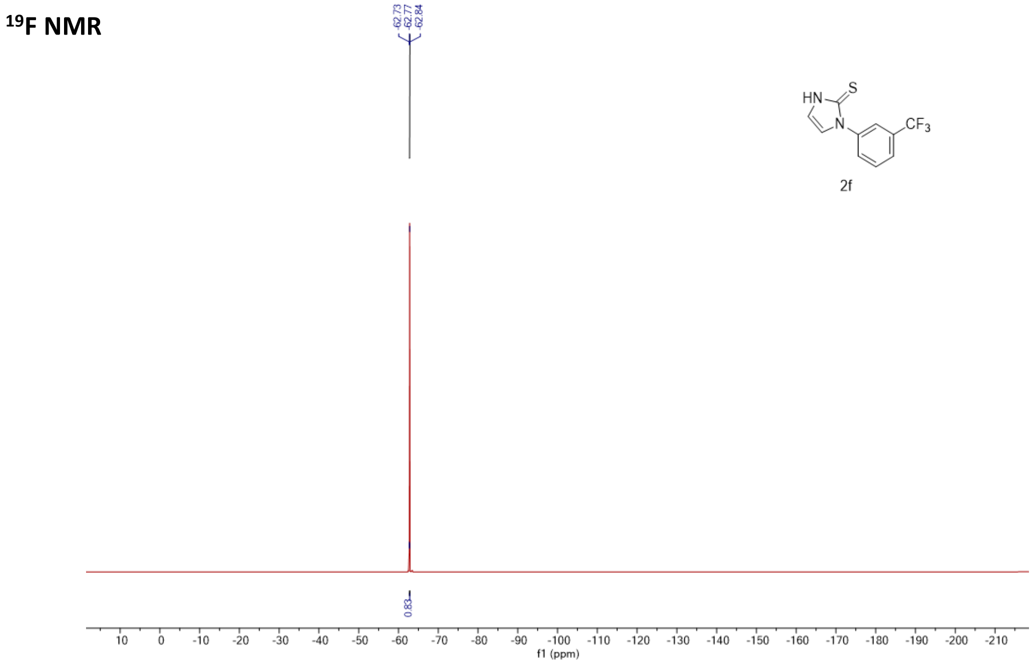

400 MHz, $CDCl_3$

^{1}H NMR

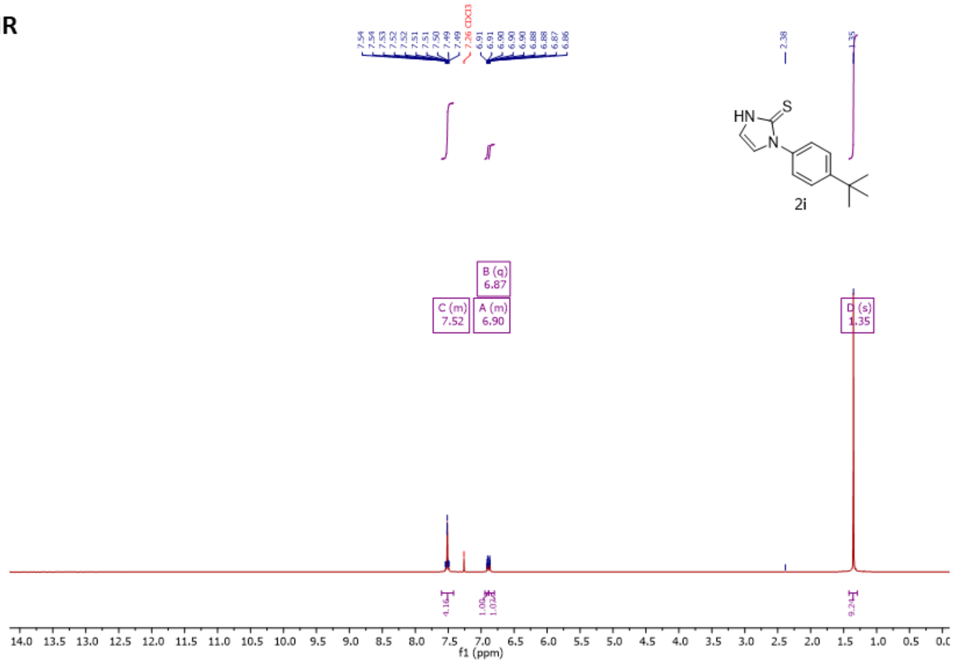

101 MHz, $CDCl_3$

^{13}C NMR

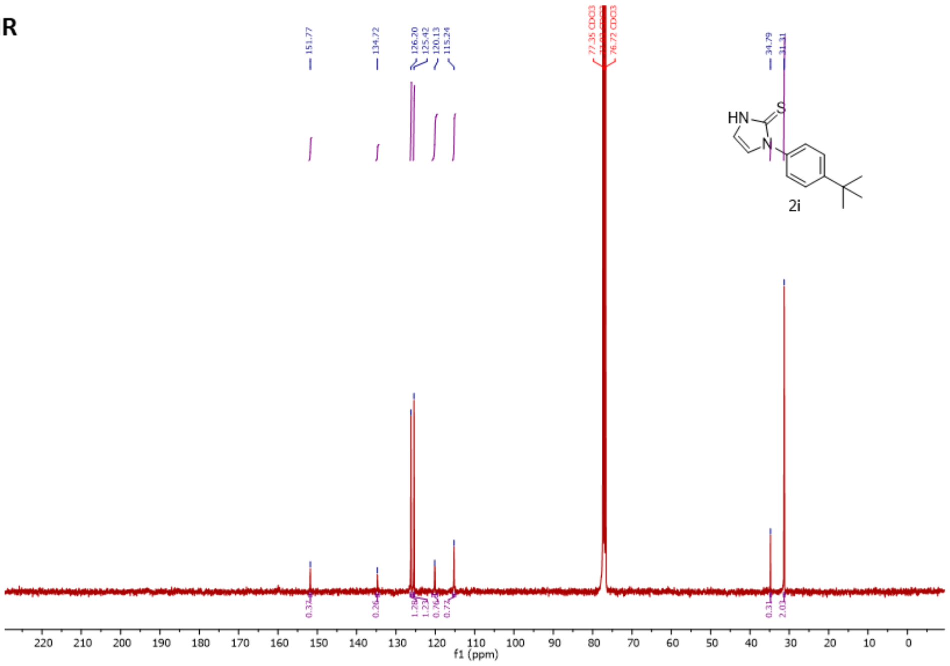

400 MHz, $CDCl_3$

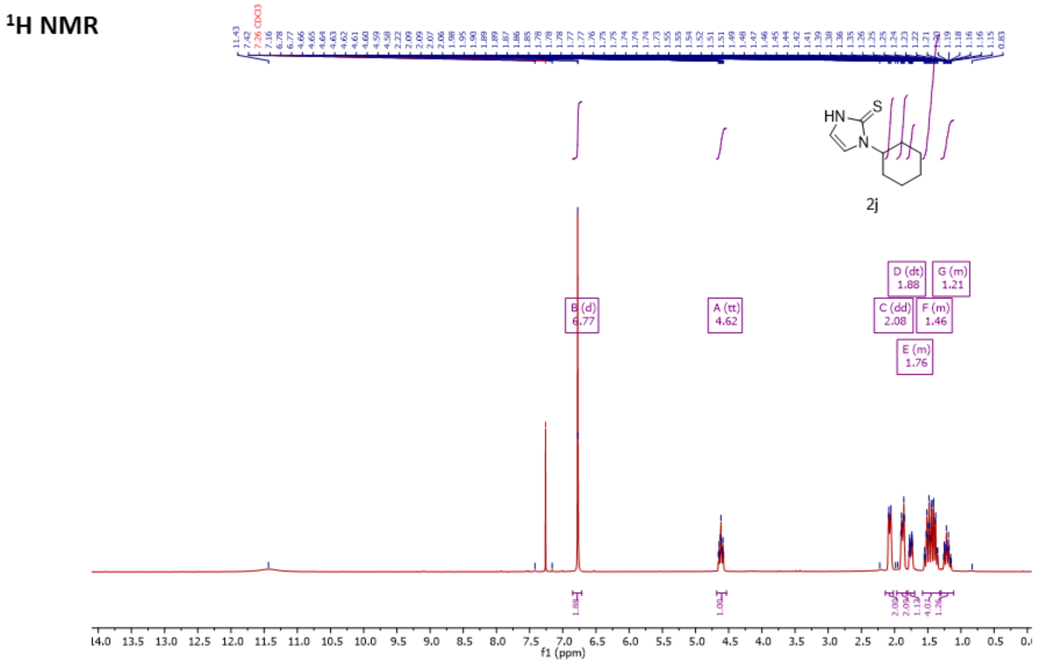

101 MHz, $CDCl_3$

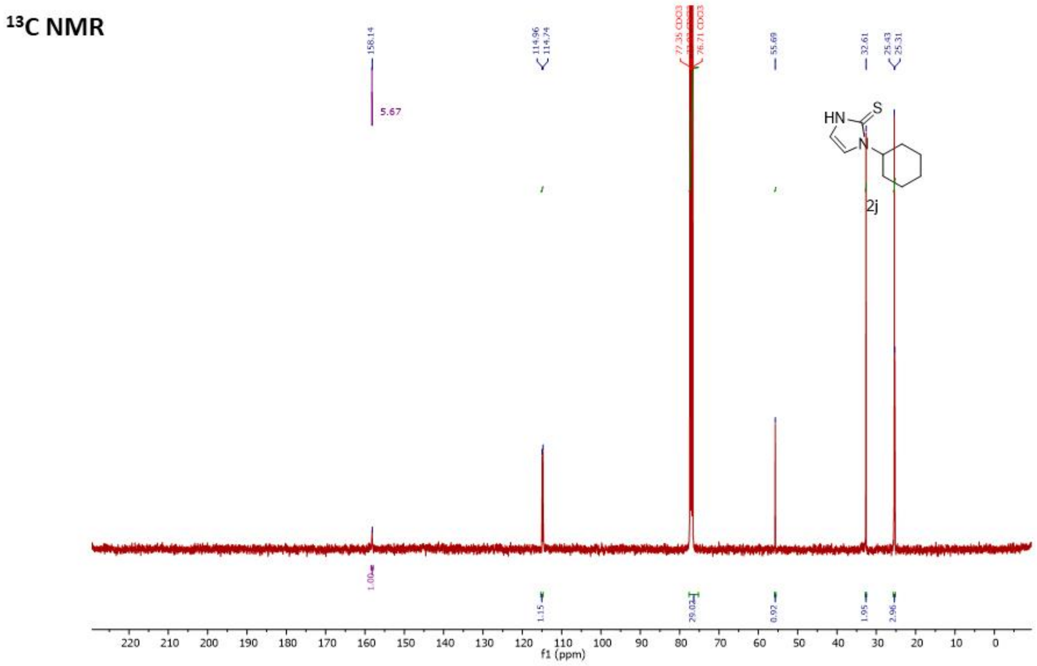

400 MHz, $CDCl_3$

^{1}H NMR

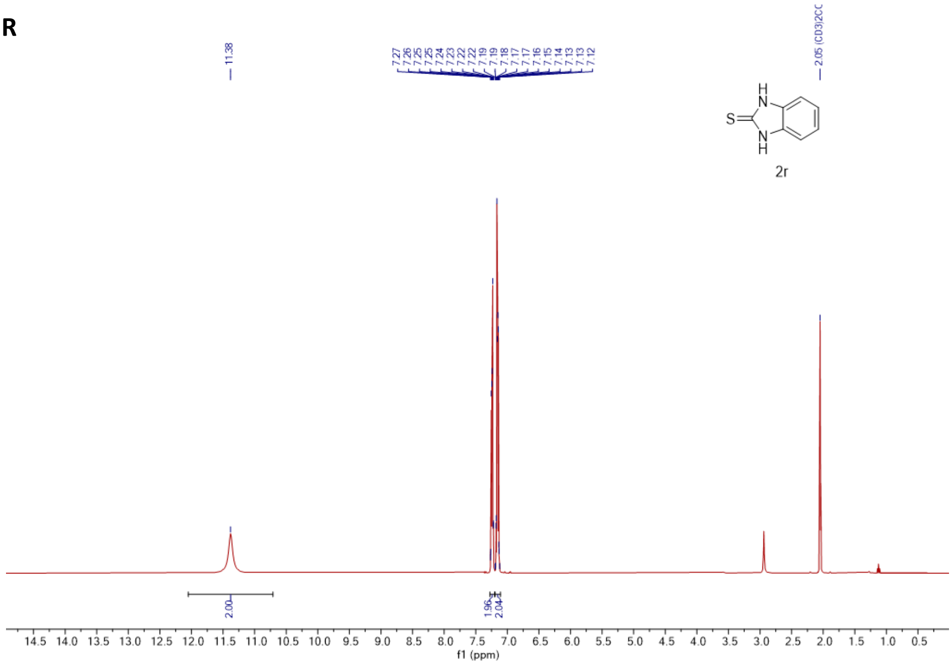

101 MHz, $CDCl_3$

^{13}C NMR

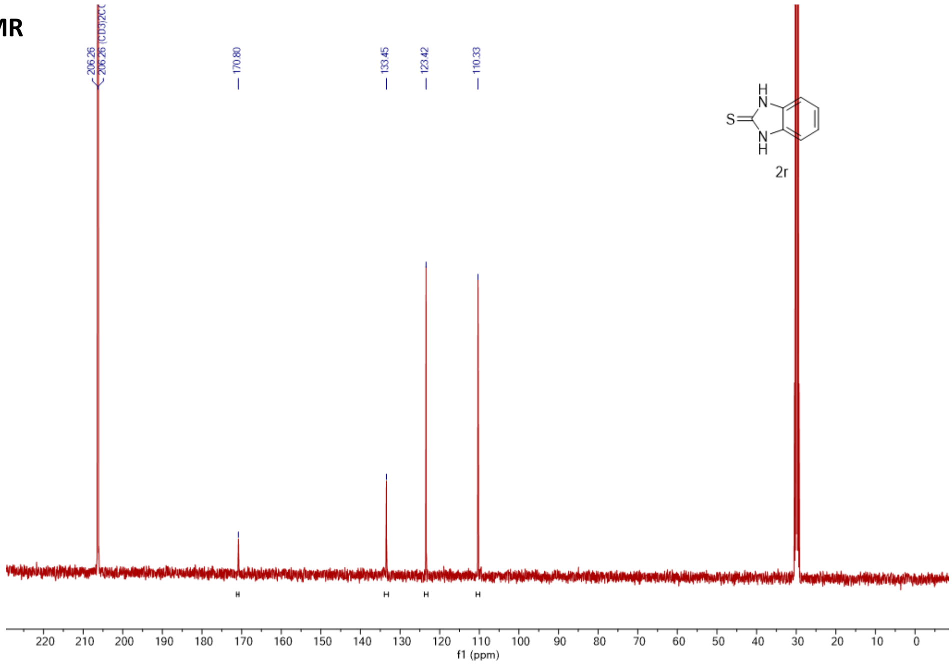

400 MHz, $CDCl_3$

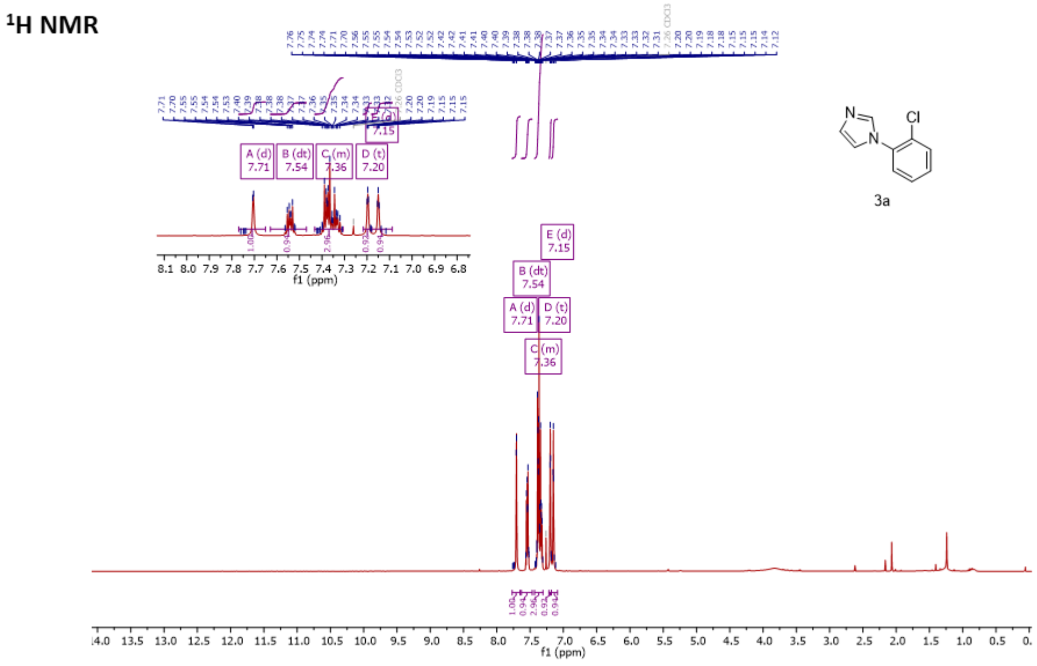

101 MHz, $CDCl_3$

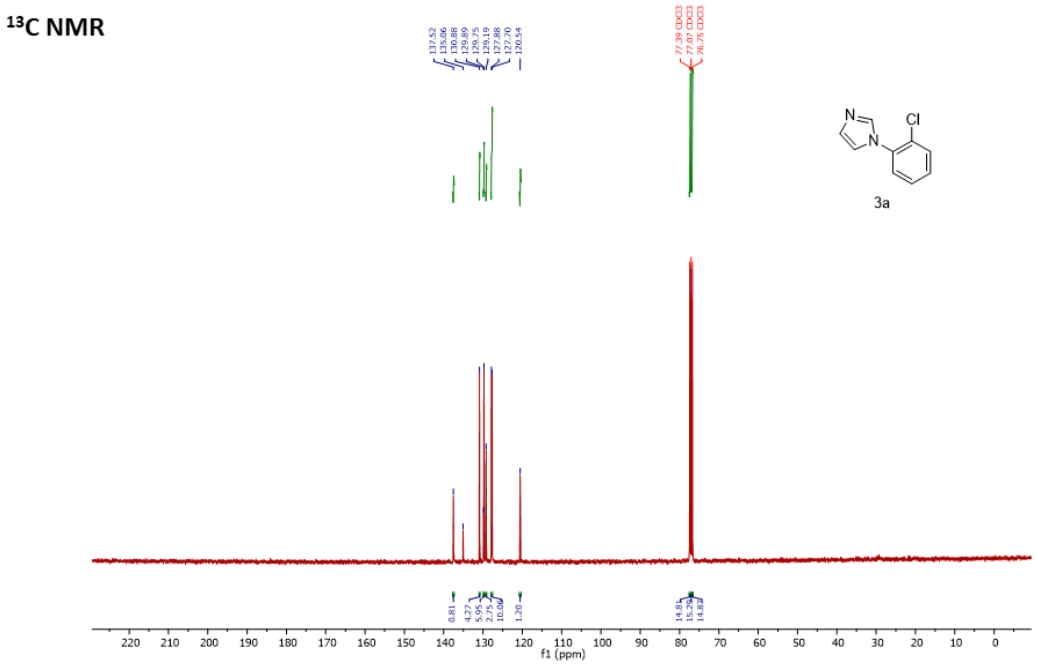

400 MHz, $CDCl_3$

^{1}H NMR

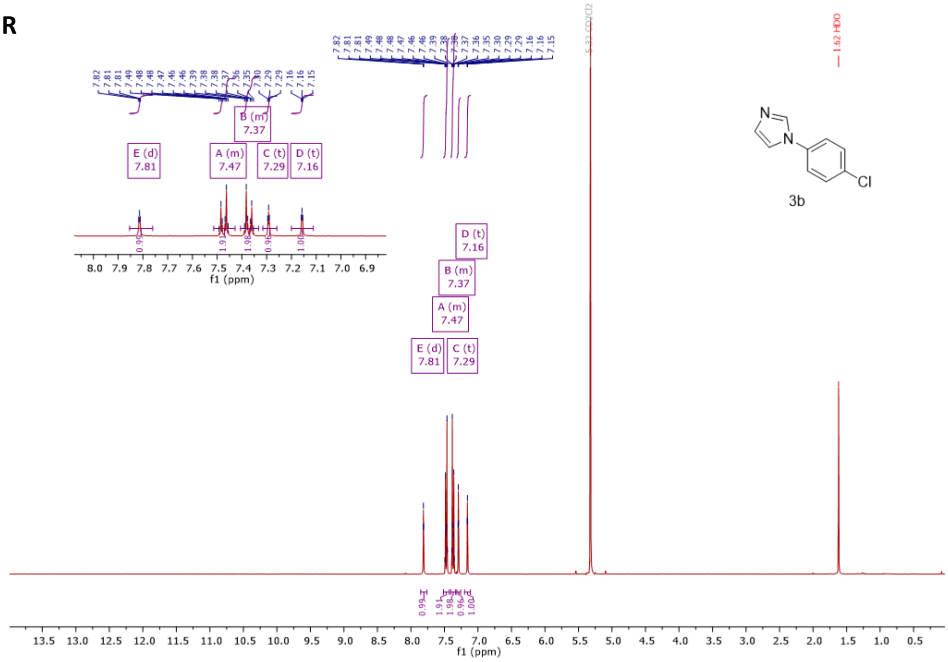

101 MHz, $CDCl_3$

^{13}C NMR

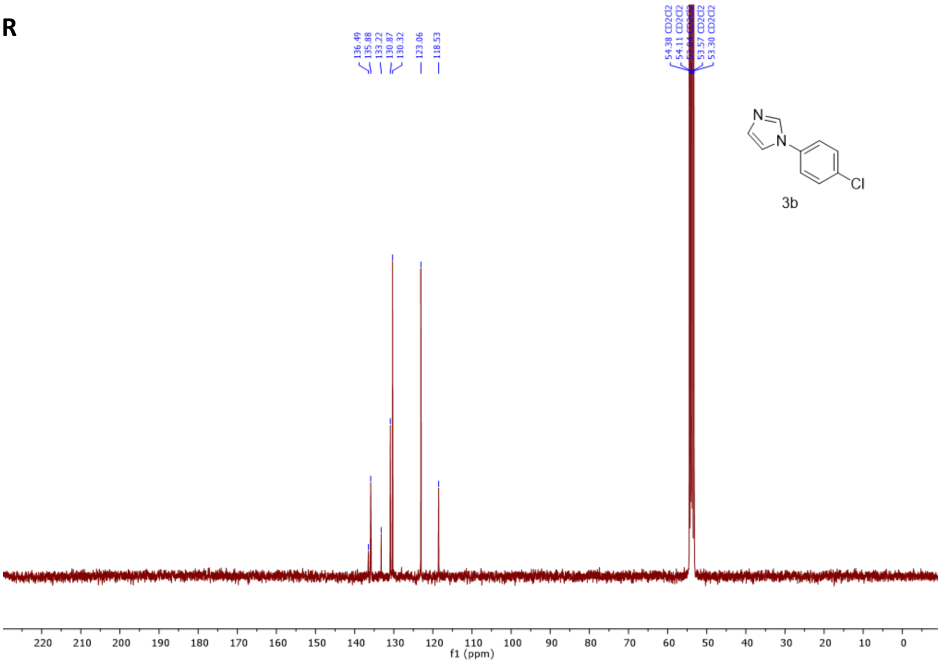

400 MHz, $CDCl_3$

^{1}H NMR

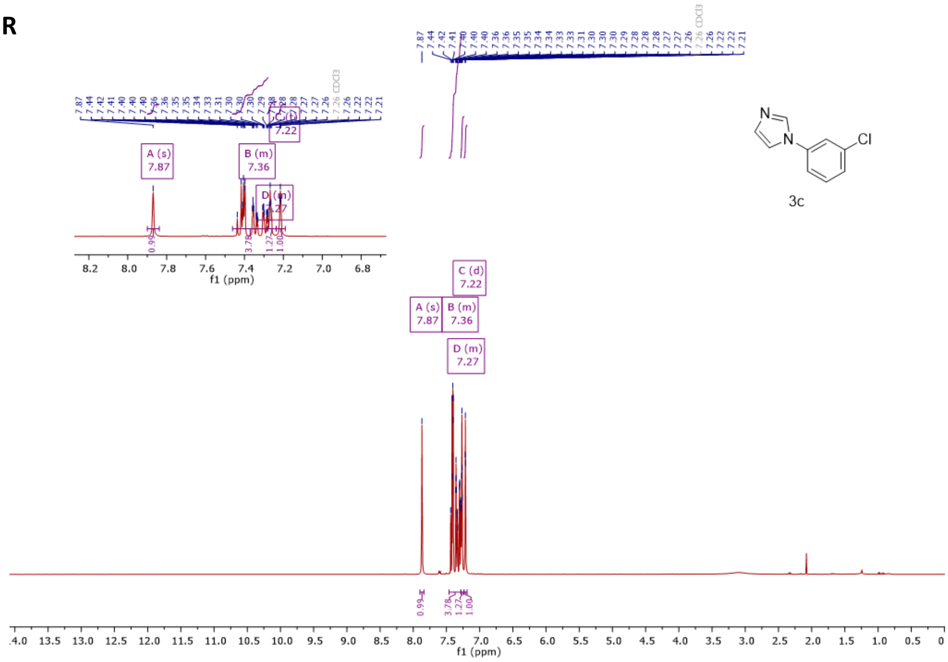

3c

101 MHz, $CDCl_3$

^{13}C NMR

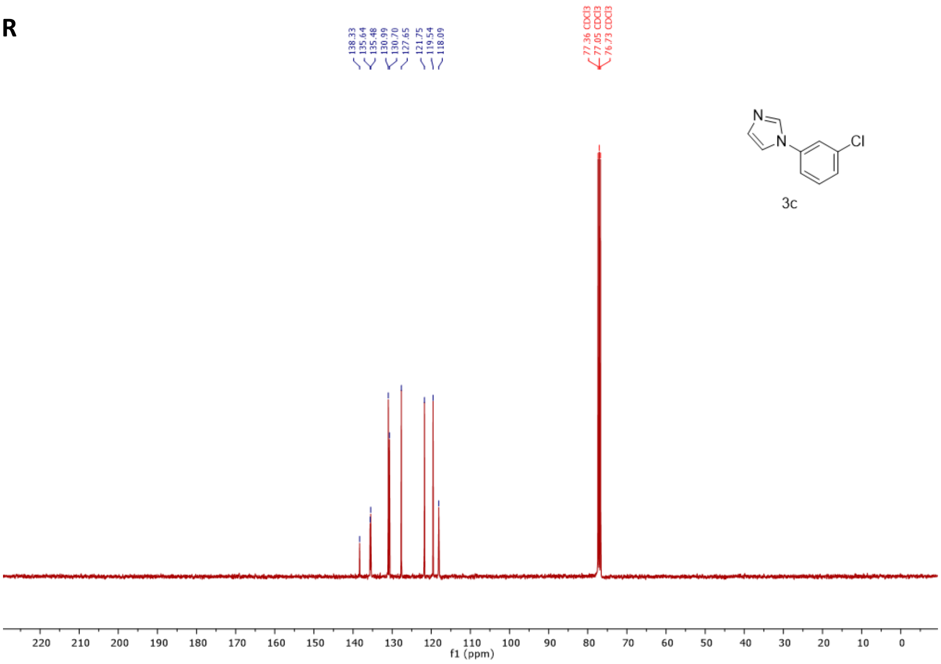

3c

400 MHz, $CDCl_3$

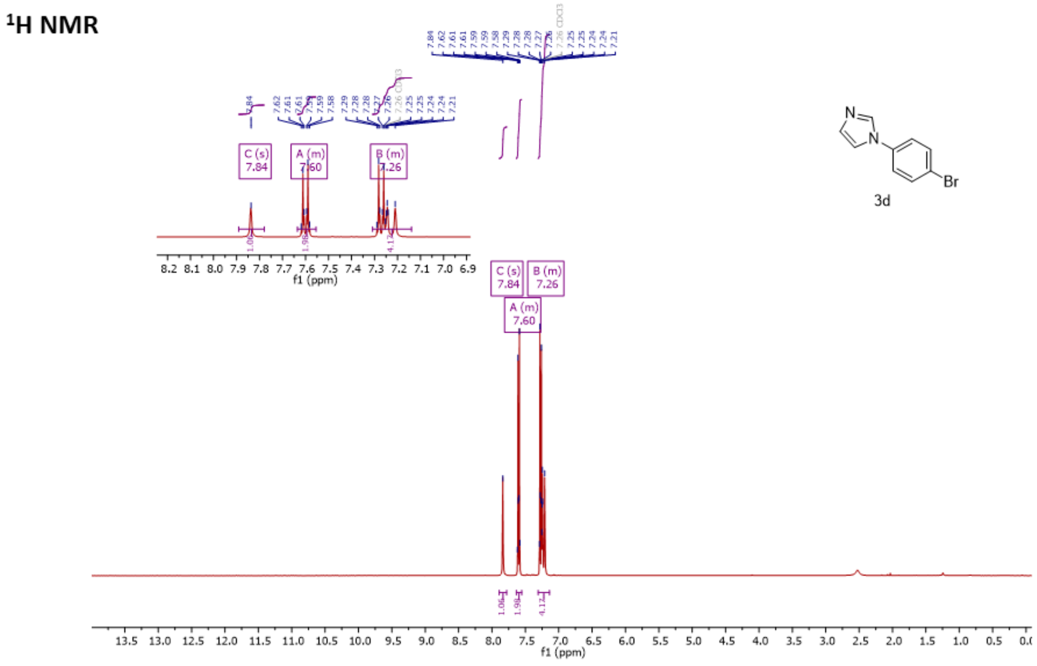

101 MHz, $CDCl_3$

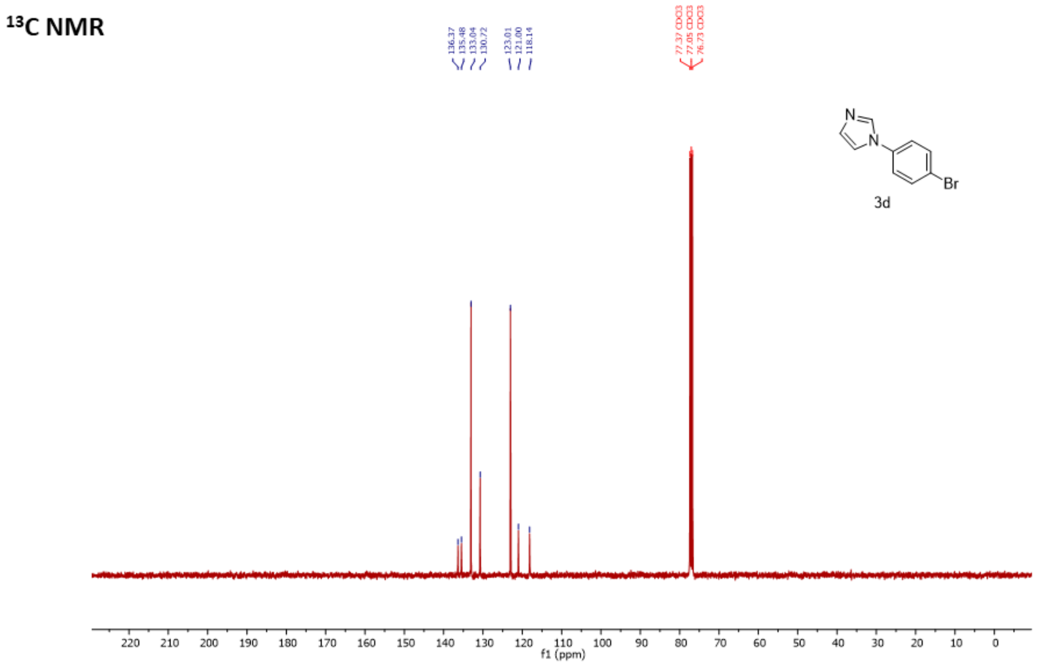

400 MHz, $CDCl_3$

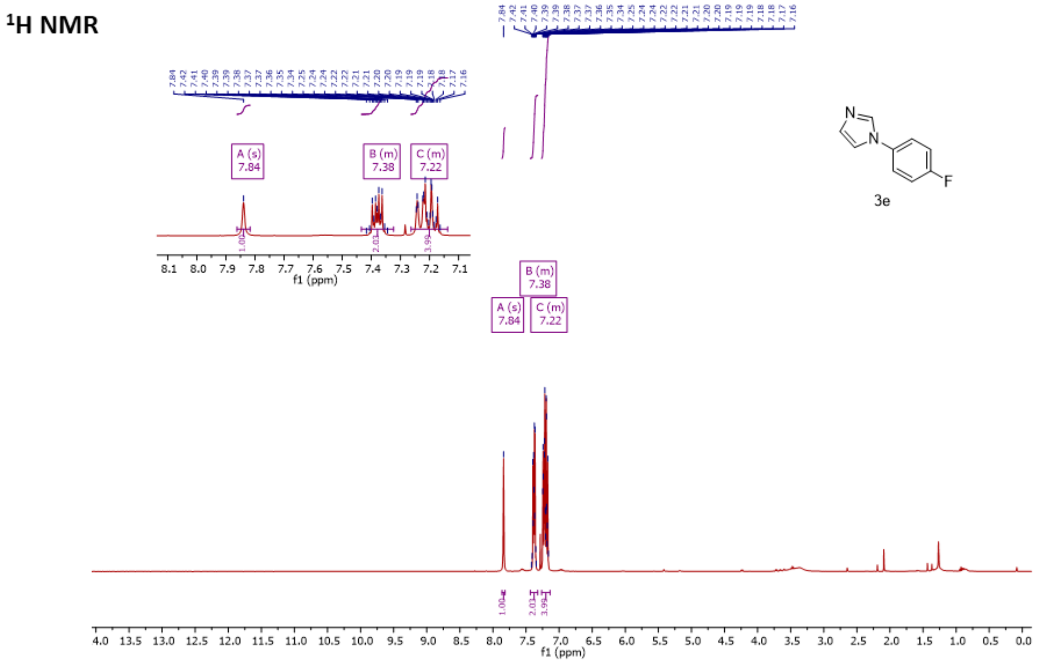

101 MHz, $CDCl_3$

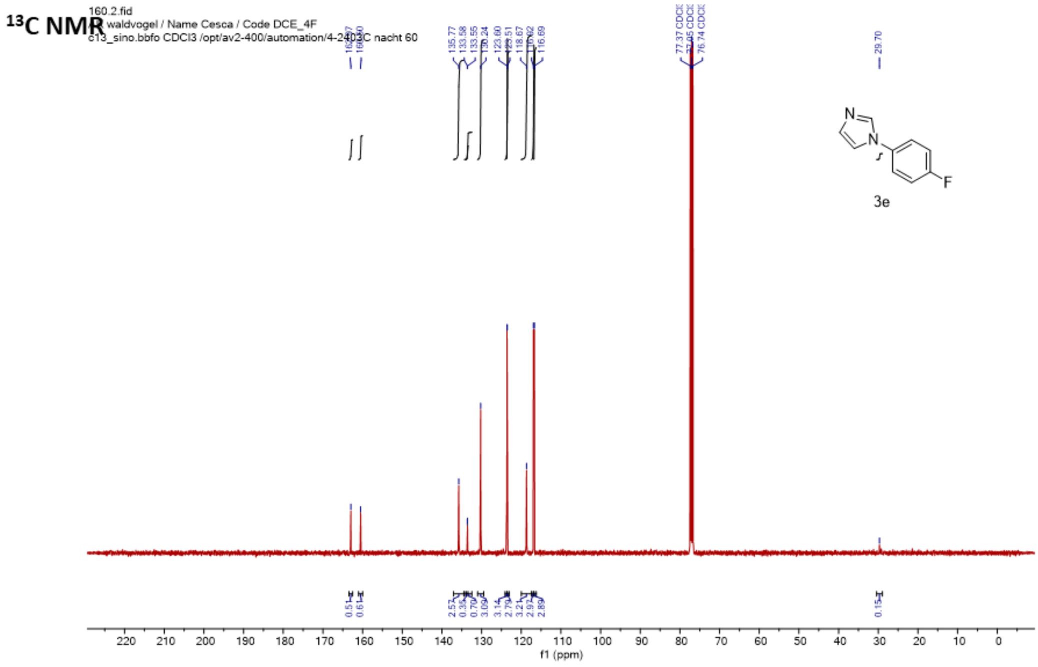

376 MHz, $CDCl_3$

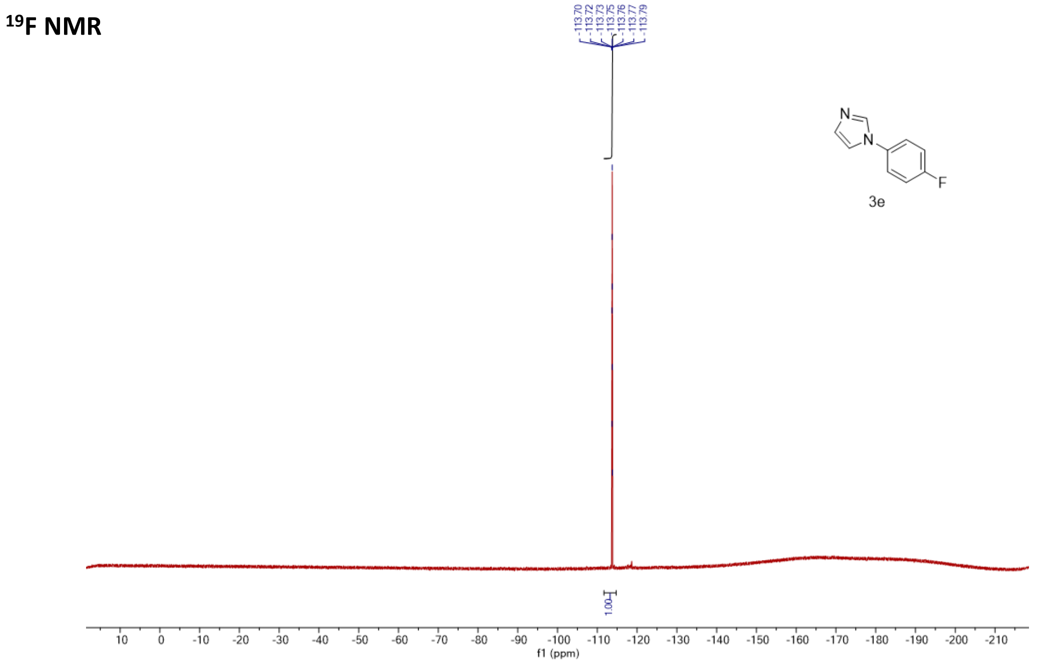

400 MHz, $CDCl_3$

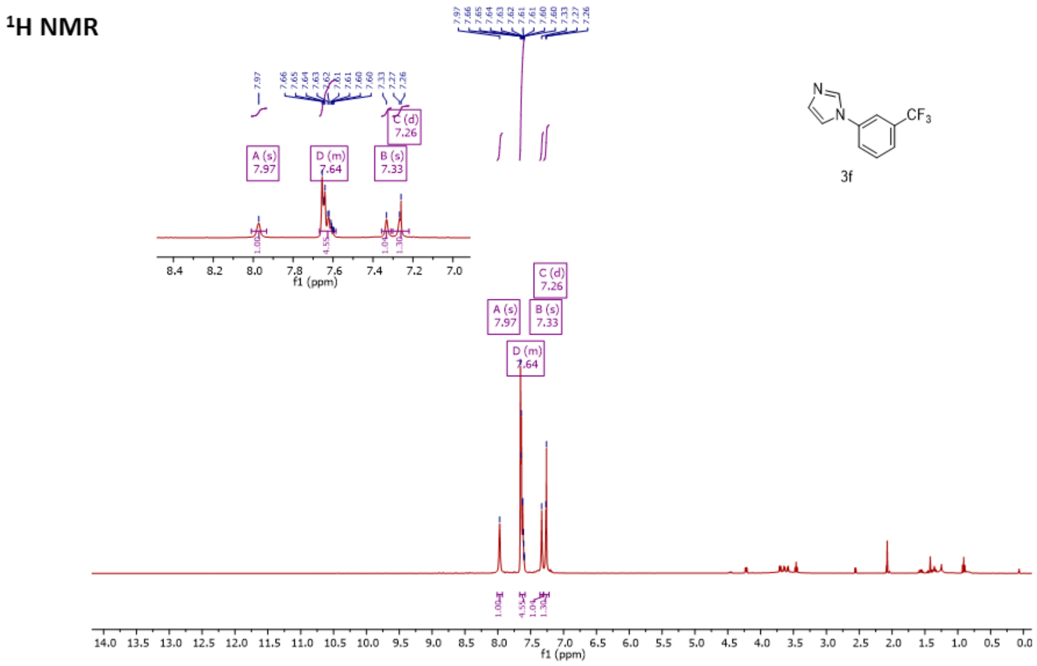

101 MHz, $CDCl_3$

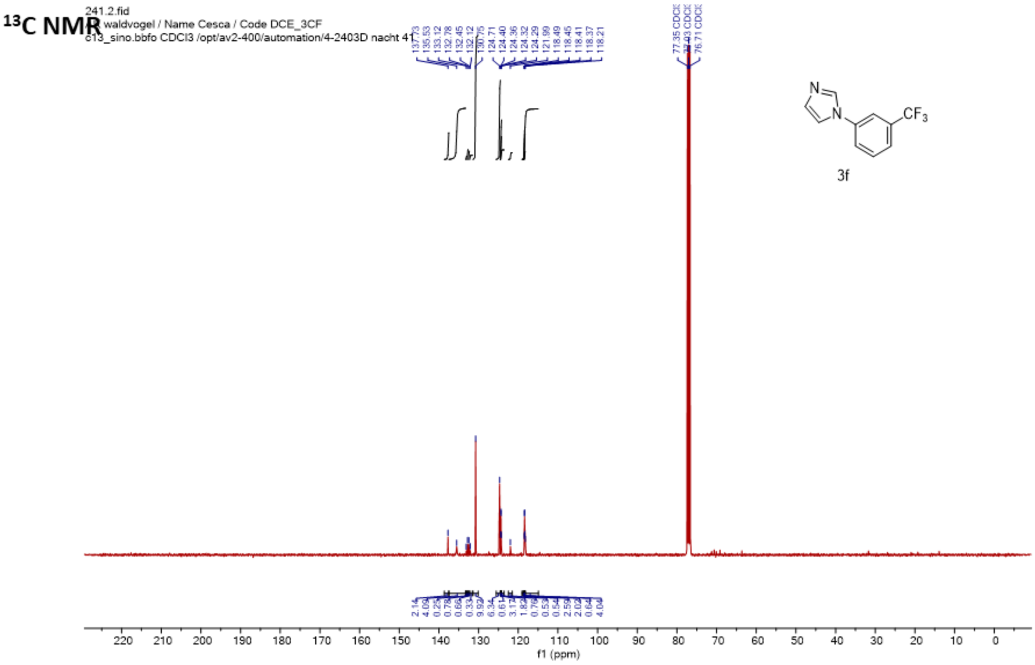

376 MHz, $CDCl_3$

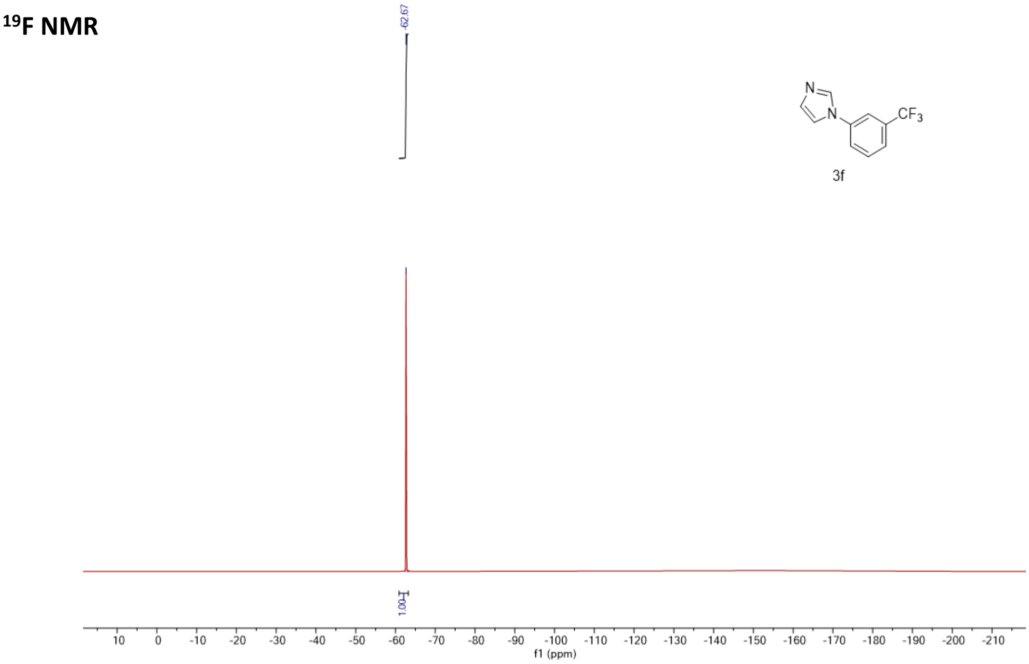

400 MHz, $CDCl_3$

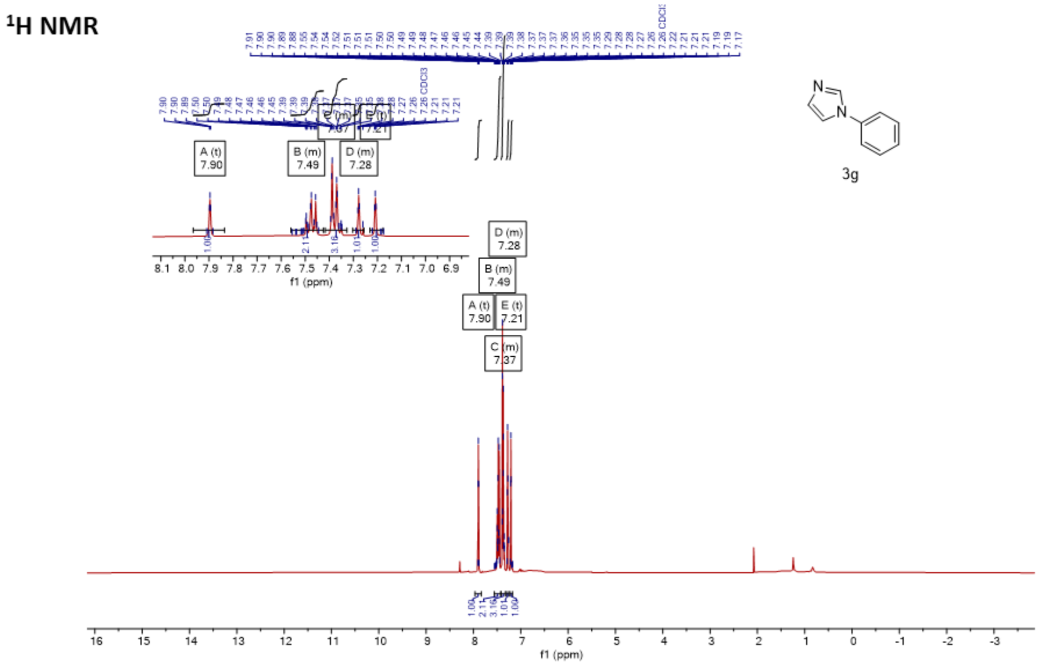

101 MHz, $CDCl_3$

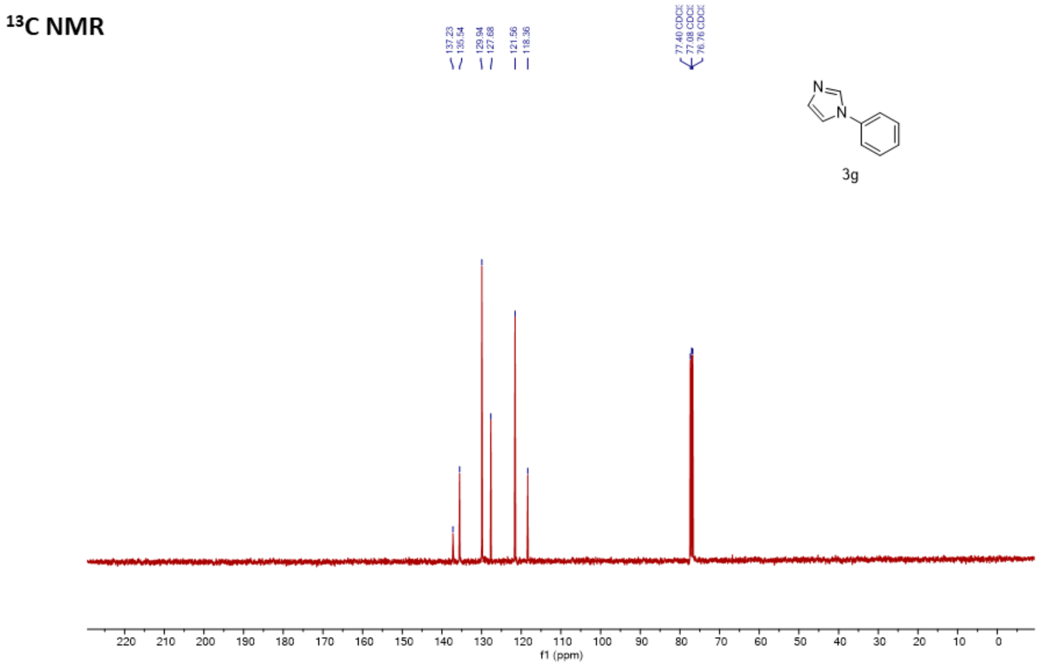

400 MHz, $CDCl_3$

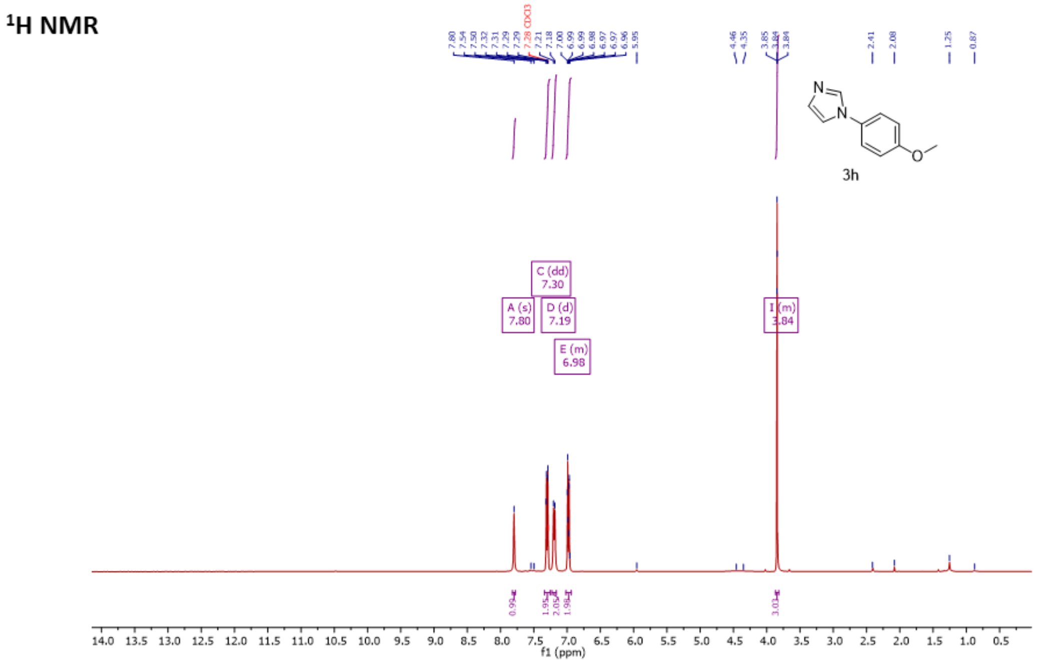

101 MHz, $CDCl_3$

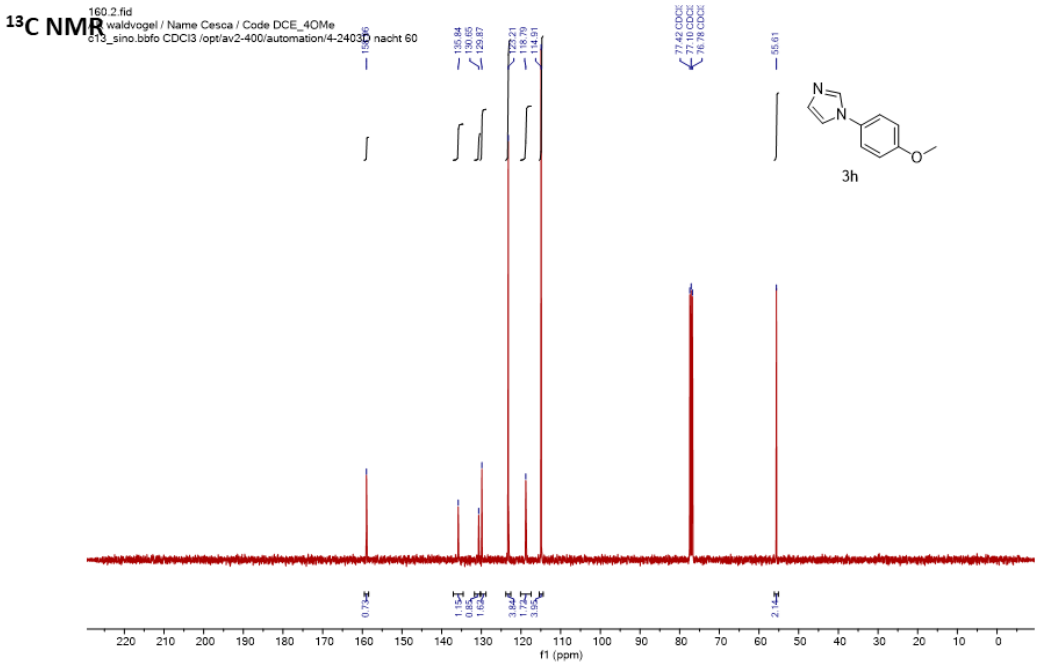

400 MHz, $CDCl_3$

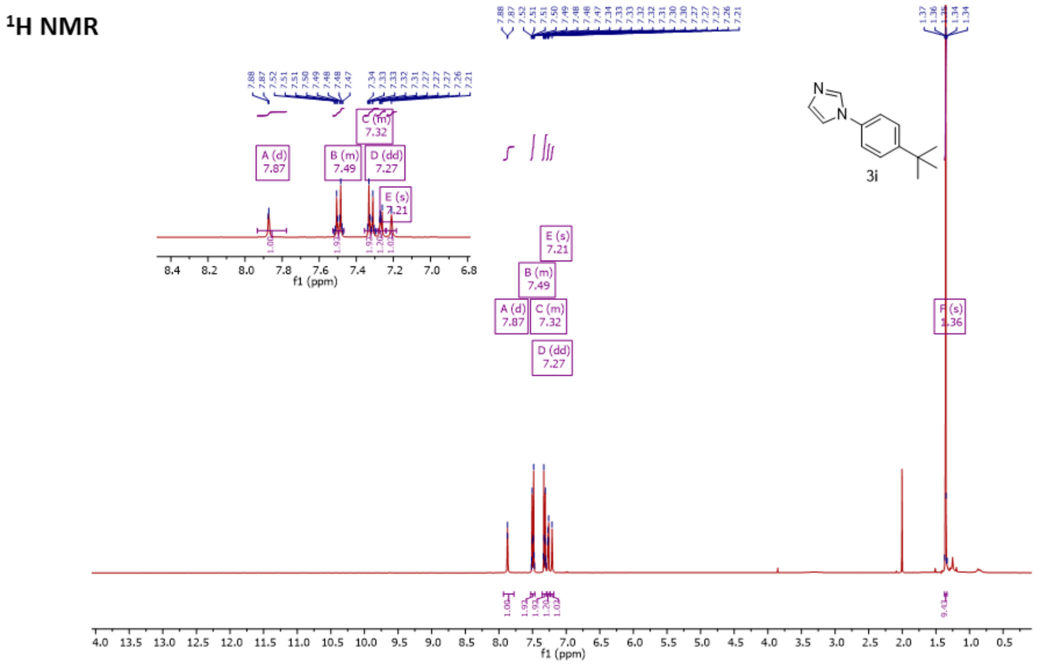

101 MHz, $CDCl_3$

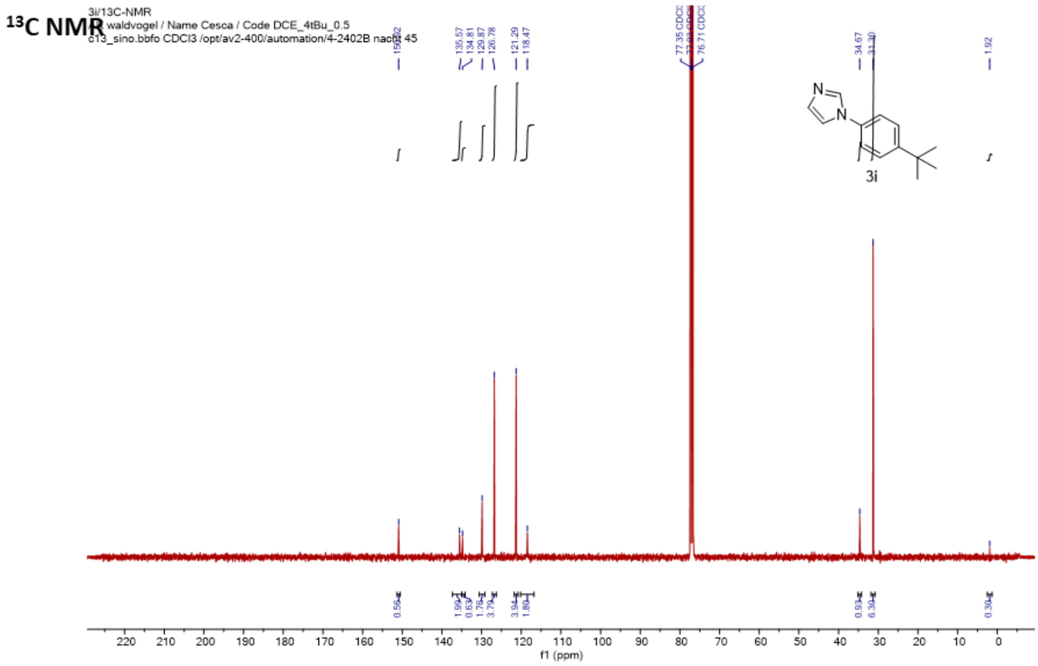

400 MHz, $CDCl_3$

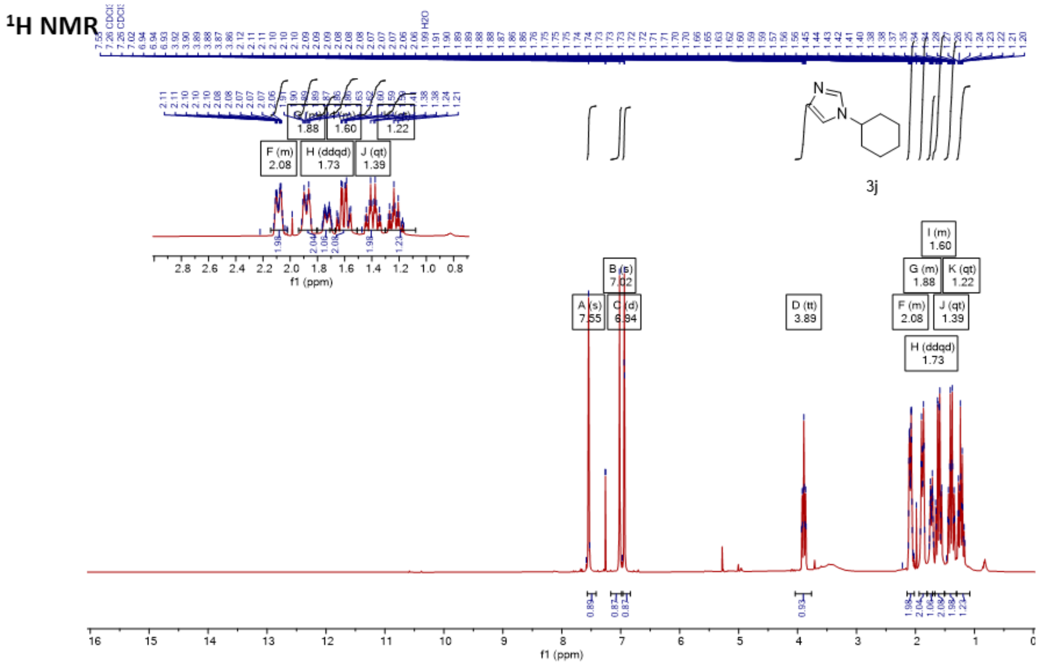

101 MHz, $CDCl_3$

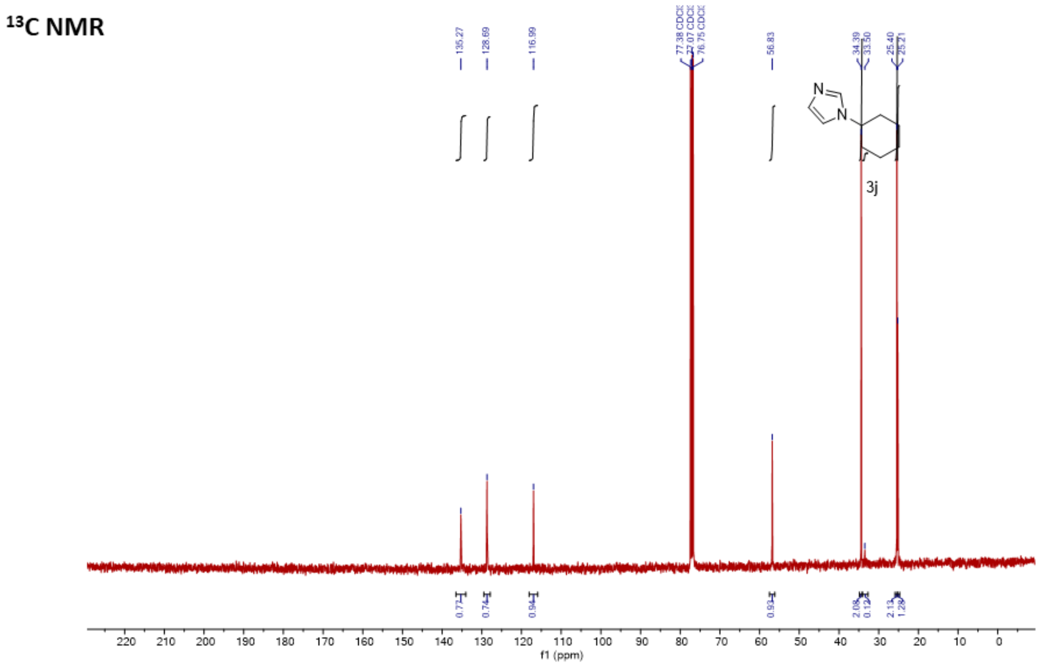

400 MHz, $CDCl_3$

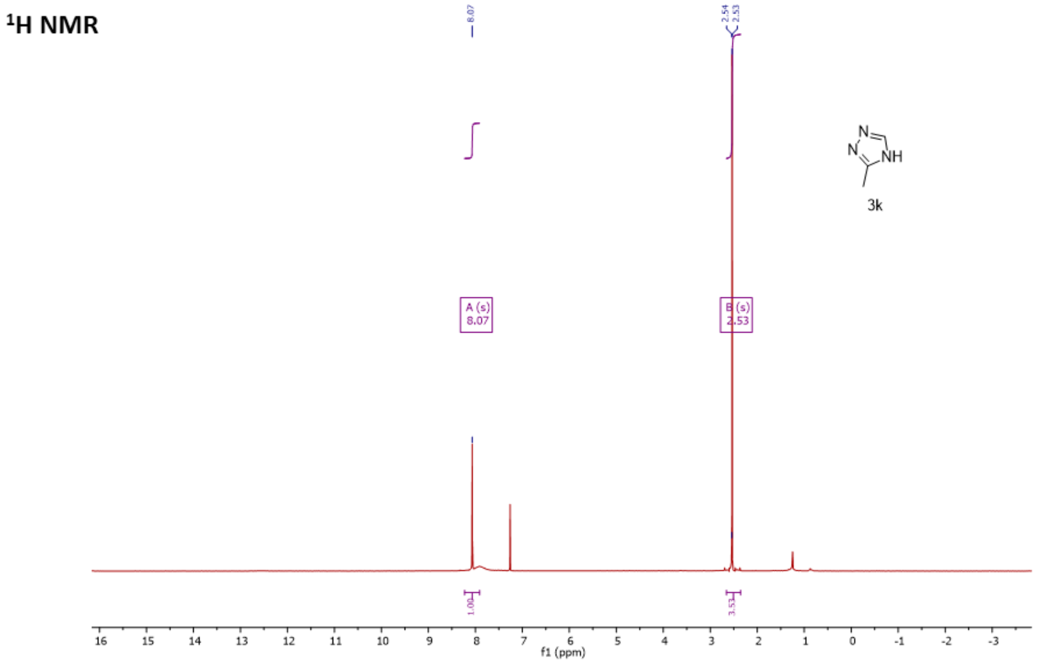

101 MHz, $CDCl_3$

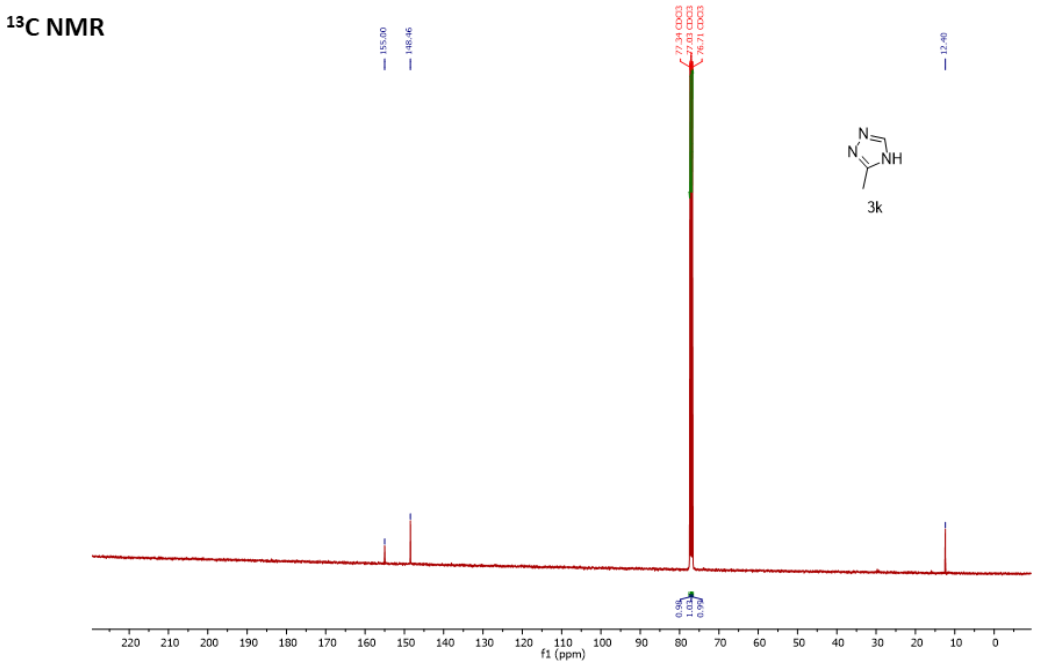

400 MHz, $CDCl_3$

^{1}H NMR

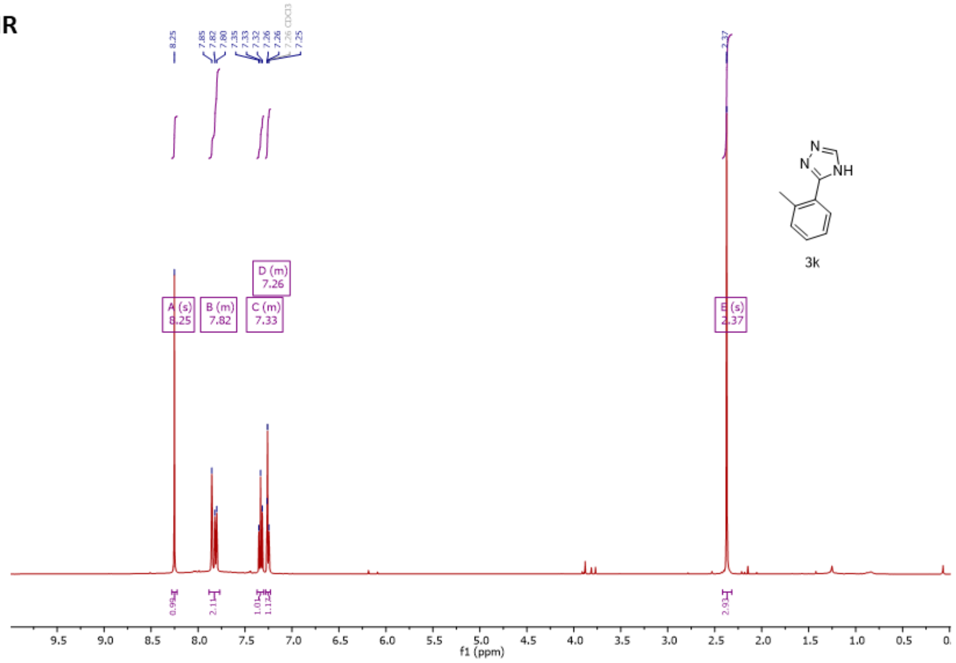

101 MHz, $CDCl_3$

^{13}C NMR

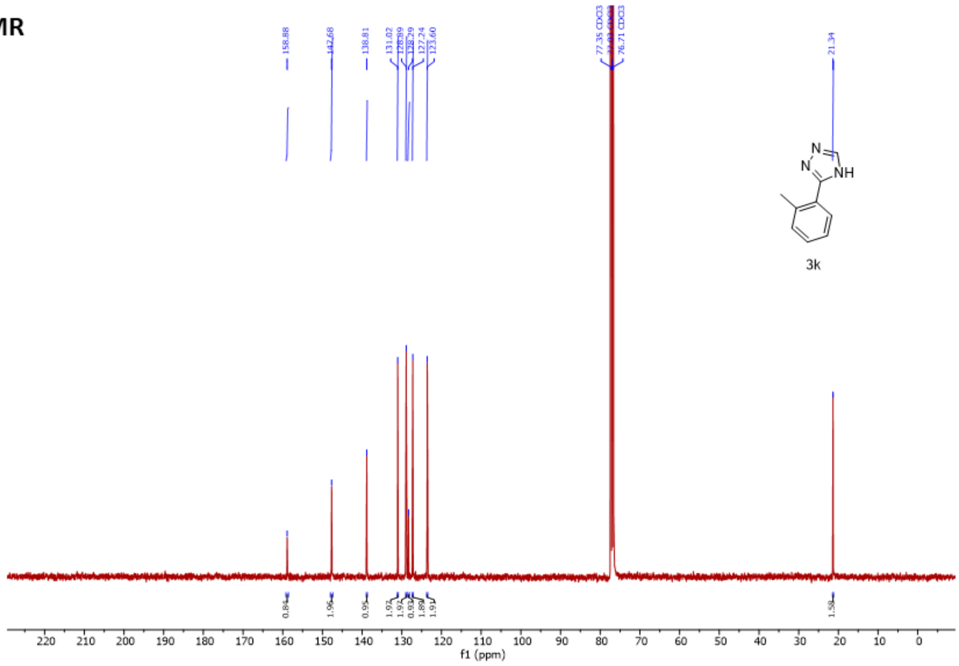

400 MHz, $CDCl_3$

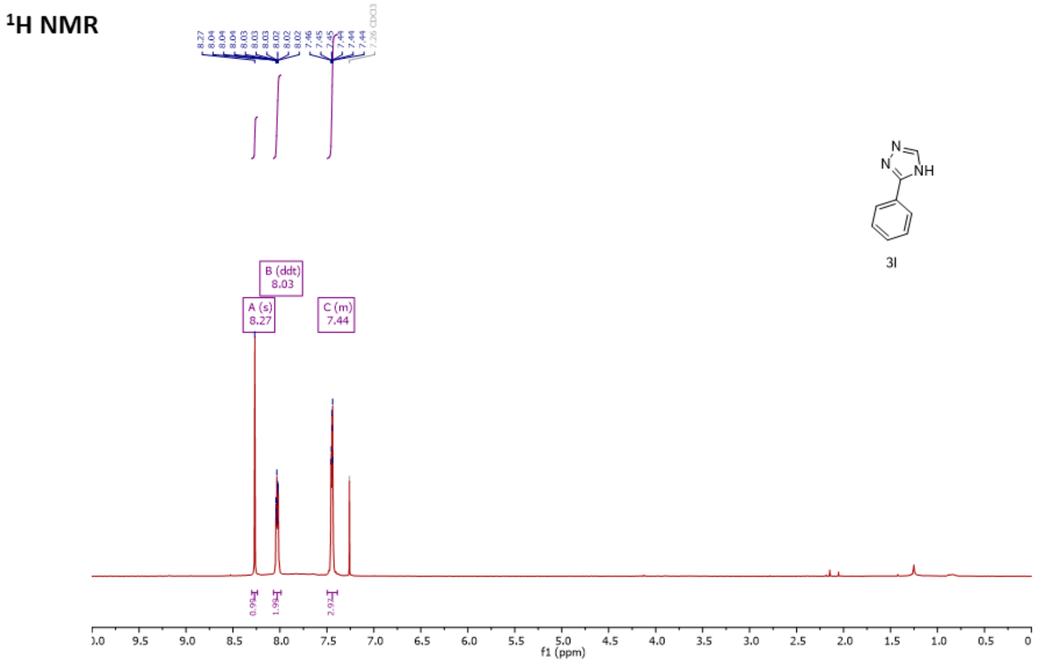

101 MHz, $CDCl_3$

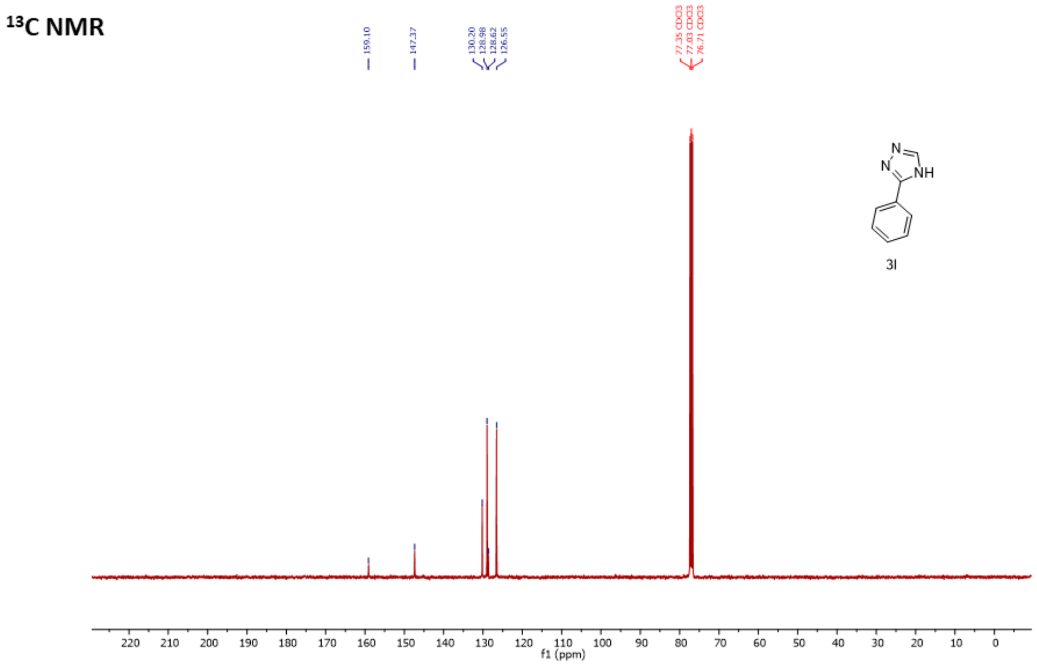

400 MHz, $CDCl_3$

^{1}H NMR

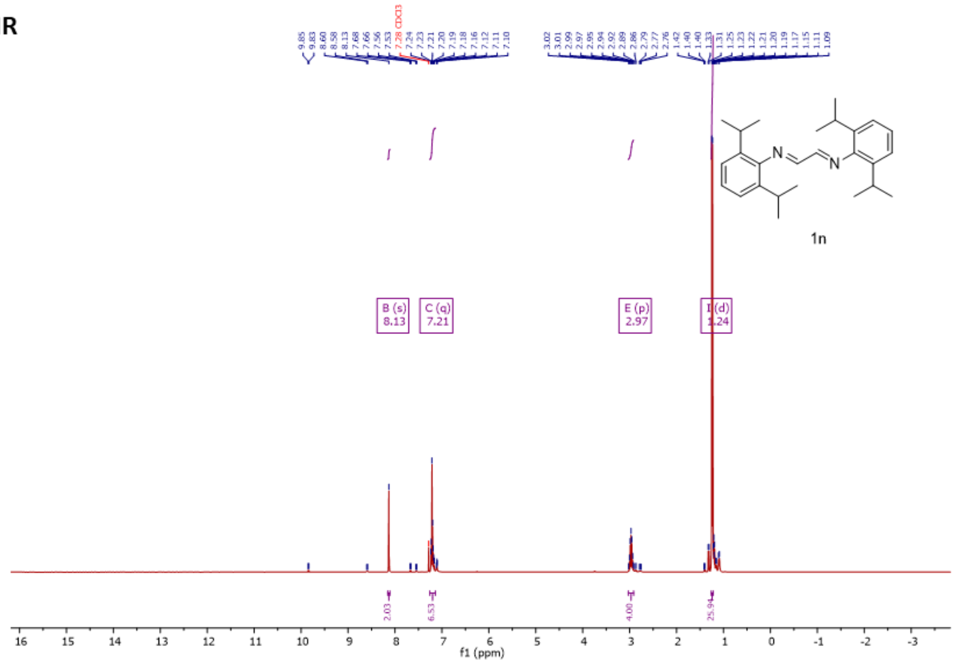

101 MHz, $CDCl_3$

^{13}C NMR

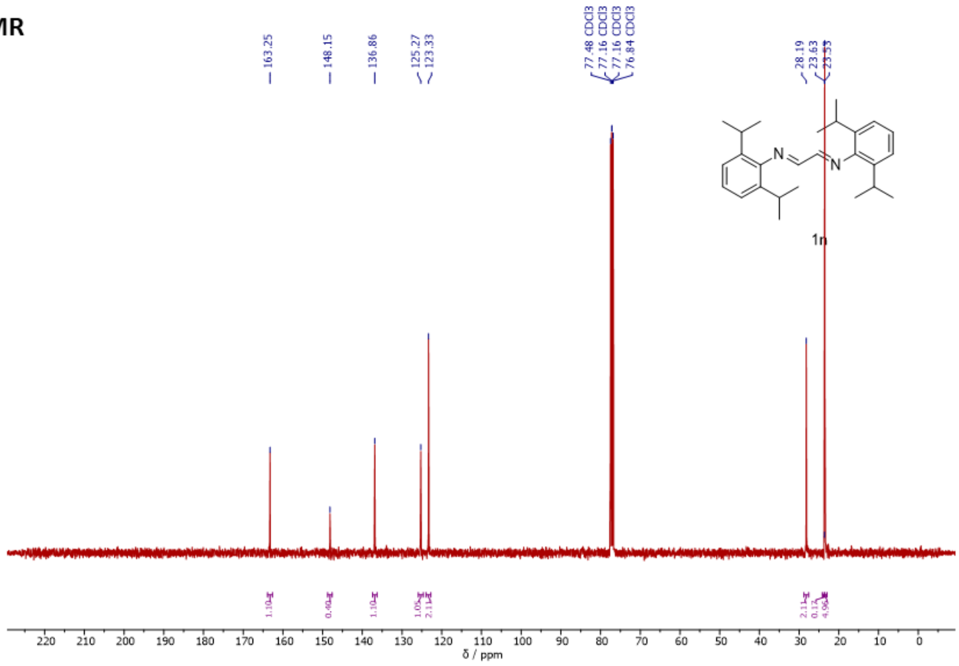

400 MHz, $CDCl_3$

^{1}H NMR

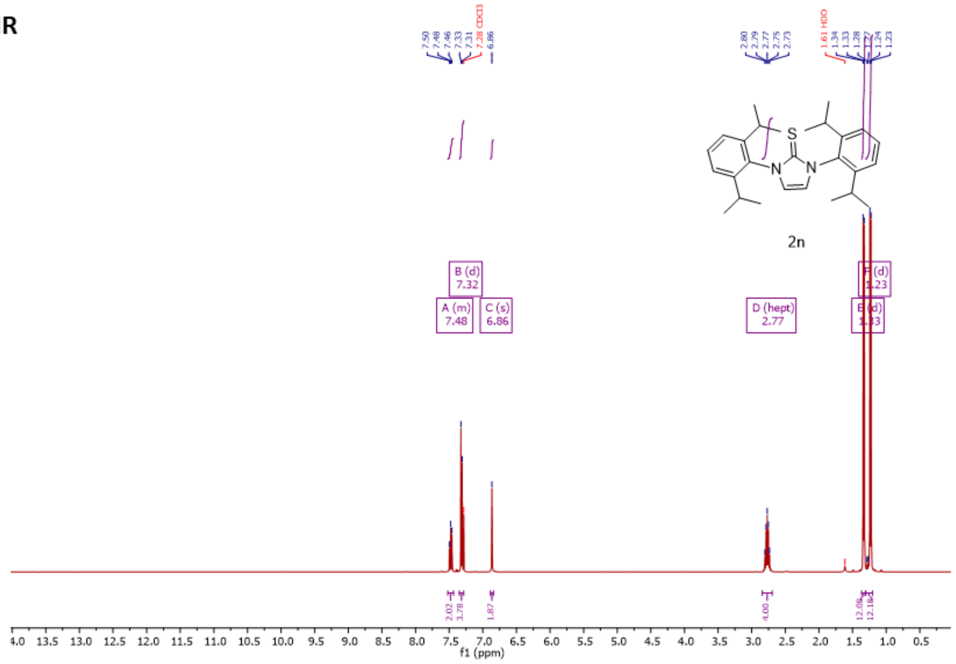

101 MHz, $CDCl_3$

^{13}C NMR

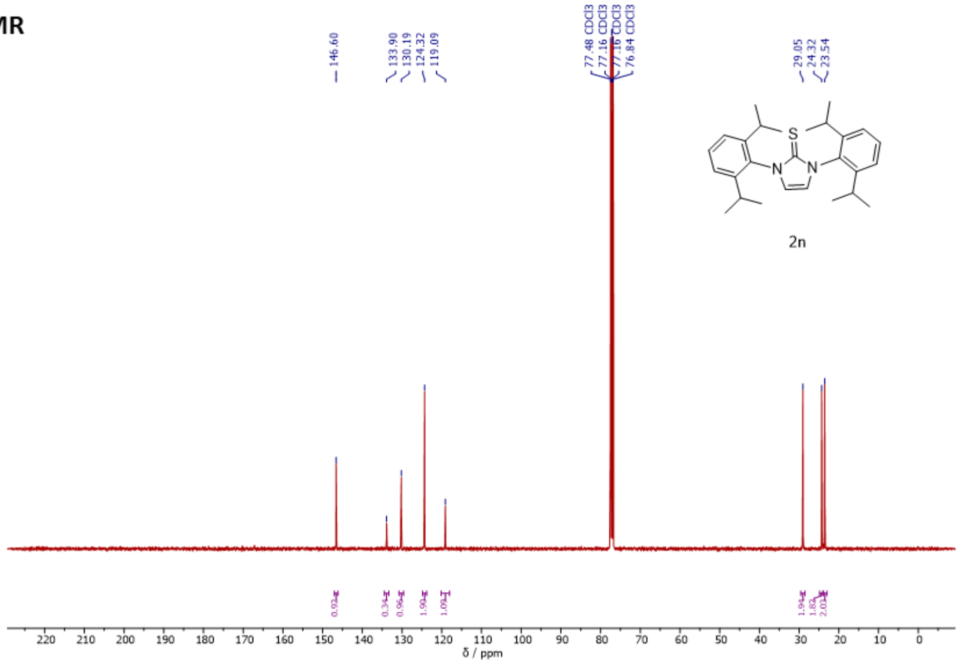

400 MHz, $CDCl_3$

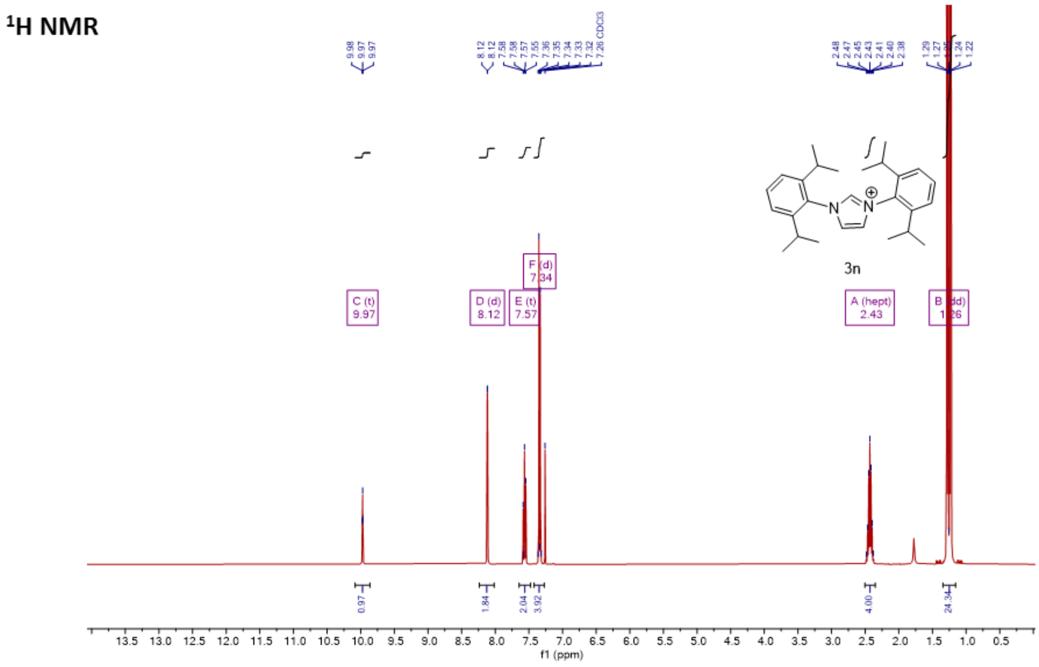

101 MHz, $CDCl_3$

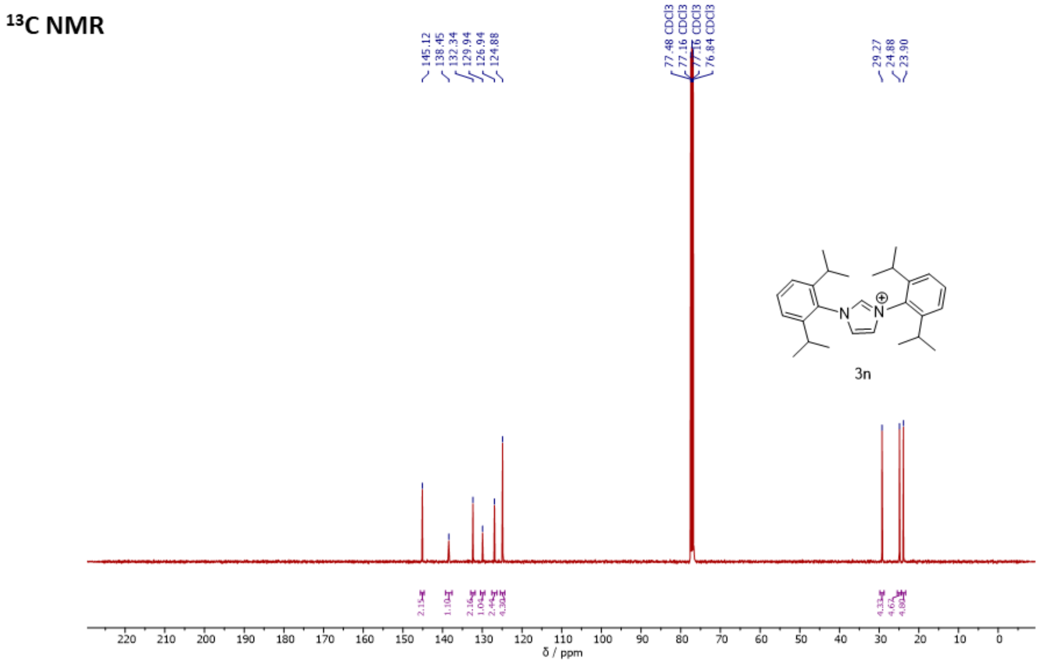

400 MHz, $CDCl_3$

^{1}H NMR

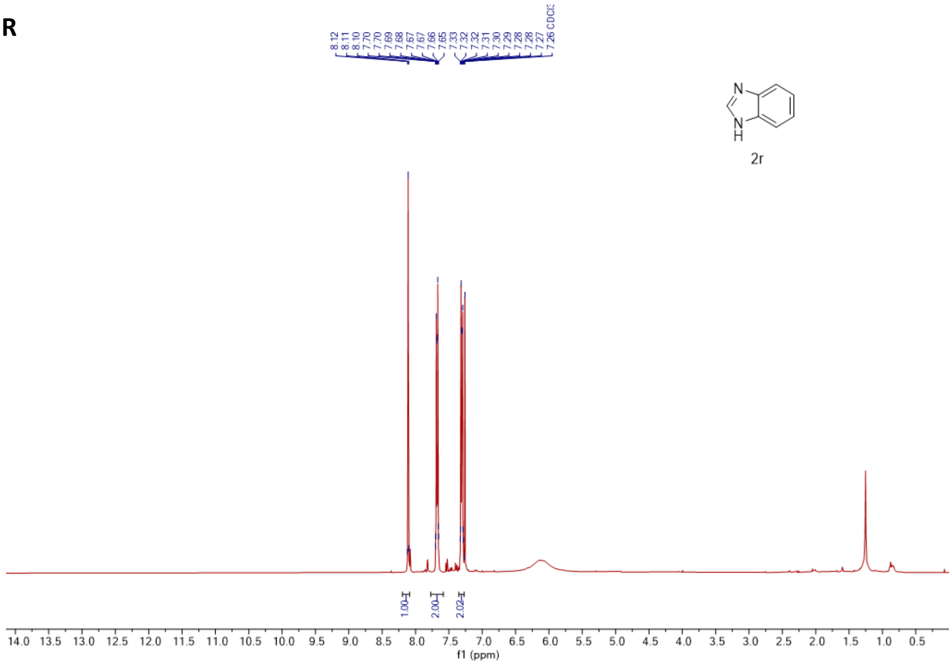

101 MHz, $CDCl_3$

^{13}C NMR

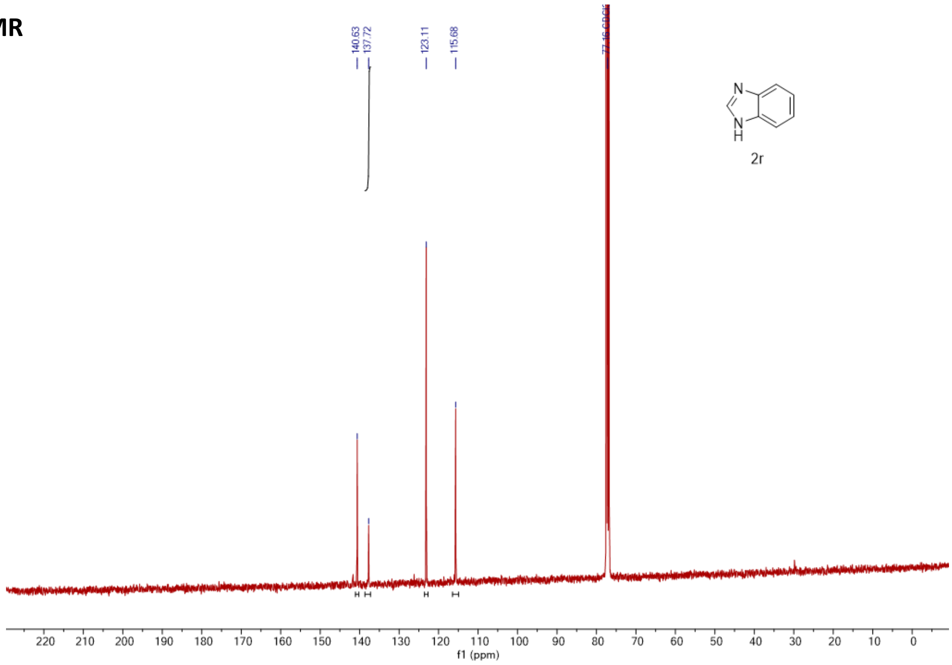

References

(49) Medvedko, S.; Ströbele, M.; Wagner, J. P. Synthesis of Sterically Encumbered Thiourea S-Oxides through Direct Thiourea Oxidation. *Chem. Eur. J.* **2023**, *29* (4), e202203005. https://doi.org/10.1002/chem.202203005.

(50) Discovery of Benzimidazole Derivatives as Modulators of Mitochondrial Function: A Potential Treatment for Alzheimer's Disease. *Eur. J. of Med. Chem*. **2017**, *125*, 1172–1192. https://doi.org/10.1016/j.ejmech.2016.11.017.

(50) Matsuda, K.; Yanagisawa, I.; Isomura, Y.; Mase, T.; Shibanuma, T. One-Pot Preparation of 1-Substituted Imidazole-2-Thione from Isothiocyanate and Amino Acetal. Synth. Commun. **1997**, *27* (20), 3565–3571. https://doi.org/10.1080/00397919708007078.

(51) Tay, N. E. S.; Nicewicz, D. A. Cation Radical Accelerated Nucleophilic Aromatic Substitution via Organic Photoredox Catalysis. *J. Am. Chem. Soc.* **2017**, *139* (45), 16100–16104. https://doi.org/10.1021/jacs.7b10076.

(52) Ferlin, F.; Trombettoni, V.; Luciani, L.; Fusi, S.; Piermatti, O.; Santoro, S.; Vaccaro, L. A Waste-Minimized Protocol for Copper-Catalyzed Ullmann-Type Reaction in a Biomass Derived Furfuryl Alcohol/Water Azeotrope. Green Chem. **2018**, *20* (7), 1634–1639. https://doi.org/10.1039/C8GC00287H.

(53) Guo, M.; Chen, B.; Chen, K.; Guo, S.; Liu, F.-S.; Xu, C.; Yao, H.-G. N-Heterocyclic Carbene Copper Complex Catalyzed Chan-Evans-Lam Reactions of Arylboronic Acids with Azoles and Amines. Tetrahedron Letters **2022**, *107*, 154074. https://doi.org/10.1016/j.tetlet.2022.154074.

(54) Sreedhar, B.; Venkanna, G. T.; Kumar, K. B. S.; Balasubrahmanyam, V. Copper(I) Oxide Catalyzed N-Arylation of Azoles and Amines with Arylboronic Acid at Room Temperature under Base-Free Conditions. Synthesis **2008**, 795–799. https://doi.org/10.1055/s-2008-1032184.

(55) Suresh, P.; Pitchumani, K. Per-6-Amino-β-Cyclodextrin as an Efficient Supramolecular Ligand and Host for Cu(I)-Catalyzed N-Arylation of Imidazole with Aryl Bromides. *J. Org. Chem.* **2008**, *73* (22), 9121–9124. https://doi.org/10.1021/jo801811w.

(56) Adhikari, B.; Teimouri, M.; Akin, J. W.; Raju, S.; Stokes, S. L.; Emerson, J. P. Cu−NHC Complex for Chan-Evans-Lam Cross-Coupling Reactions of N-Heterocyclic Compounds and Arylboronic Acids. *Eur J Org Chem* **2023**, *26* (40), e202300620. https://doi.org/10.1002/ejoc.202300620.

(57) Xia, R.; Sun, L.-P.; Qu, G.-R. The Synthesis of Nebularine and Its Analogs via Oxidative Desulfuration in Aqueous Nitric Acid. Phosphorus, Sulfur Silicon Relat Elem **2017**, 1*92* (1), 88–91. https://doi.org/10.1080/10426507.2016.1225057.

(58) Fu, M.; Ji, X.; Li, Y.; Deng, G.-J.; Huang, H. Visible-Light-Induced Aerobic Oxidative Desulfurization of 2-Mercaptobenzimidazoles via a Sulfinyl Radical. Green Chem. **2020**, *22* (17), 5594–5598. https://doi.org/10.1039/D0GC02269A.
